# Stochastic neural field equations: a rigorous footing

**DOI:** 10.1007/s00285-014-0807-6

**Published:** 2014-07-29

**Authors:** O. Faugeras, J. Inglis

**Affiliations:** 1 NeuroMathComp, INRIA, Sophia Antipolis, France; 2 ToSCA/NeuroMathComp, INRIA, Sophia Antipolis, France

**Keywords:** Stochastic neural field equations, Spatially correlated noise, Multiplicative noise, Stochastic integro-differential equation, Existence and uniqueness, 60H20, 60H30, 92C20

## Abstract

We here consider a stochastic version of the classical neural field equation that is currently actively studied in the mathematical neuroscience community. Our goal is to present a well-known rigorous probabilistic framework in which to study these equations in a way that is accessible to practitioners currently working in the area, and thus to bridge some of the cultural/scientific gaps between probability theory and mathematical biology. In this way, the paper is intended to act as a reference that collects together relevant rigorous results about notions of solutions and well-posedness, which although may be straightforward to experts from SPDEs, are largely unknown in the neuroscientific community, and difficult to find in a very large body of literature. Moreover, in the course of our study we provide some new specific conditions on the parameters appearing in the equation (in particular on the neural field kernel) that guarantee the existence of a solution.

## Introduction

Neural field equations have been widely used to study spatiotemporal dynamics of cortical regions. Arising as continuous spatial limits of discrete models, they provide a step towards an understanding of the relationship between the macroscopic spatially structured activity of densely populated regions of the brain, and the underlying microscopic neural circuitry. The discrete models themselves describe the activity of a large number of individual neurons with no spatial dimensions. Such neural mass models have been proposed by Lopes da Silva et al. (1974, 1976) to account for oscillatory phenomena observed in the brain, and were later put on a stronger mathematical footing in the study of epileptic-like seizures in Jansen and Rit (1995). When taking the spatial limit of such discrete models, one typically arrives at a nonlinear integro-differential equation, in which the integral term can be seen as a nonlocal interaction term describing the spatial distribution of synapses in a cortical region. Neural field models build on the original work of Wilson and Cowan (1972, Wilson and Cowan (1973)) and Amari (1977), and are known to exhibit a rich variety of phenomena including stationary states, traveling wave fronts, pulses and spiral waves. For a comprehensive review of neural field equations, including a description of their derivation, we refer to Bressloff (2012).

More recently several authors have become interested in stochastic versions of neural field equations (see for example Bressloff 2009, 2010; Bressloff and Webber 2012; Bressloff and Wilkerson 2012; Kilpatrick and Ermentrout 2013), in order to (amongst other things) model the effects of fluctuations on wave front propagation. In particular, in Bressloff and Webber (2012) a multiplicative stochastic term is added to the neural field equation, resulting in a stochastic nonlinear integro-differential equation of the form

1.1 $$\begin{aligned} dY(t, x) = \left[ - Y(t, x) + \int _{{\mathbb {R}} } w(x,y)G(Y(t, y))dy\right] dt + \sigma (Y(t, x))dW(t, x),\nonumber \\ \end{aligned}$$

for $x\in {\mathbb {R}} , t\ge 0$, and some functions $G$ (referred to as the nonlinear gain function), $\sigma$ (the diffusion coefficient) and $w$ (the neural field kernel, sometimes also called the connectivity function). Here $(W(t, x))_{x\in {\mathbb {R}} , t\ge 0}$ is a stochastic process (notionally a “Gaussian random noise”) that depends on both space and time, and which may possess some spatial correlation.

Of course the first step towards understanding (1.1) rigorously is defining what we mean by a solution. This is in fact not completely trivial and is somewhat glossed-over in the neuroscientific literature. The main point is that any solution must involve an object of the form

1.2 $$\begin{aligned} \int \sigma (Y(t, x))dW(t, x) \end{aligned}$$

which must be precisely defined. Of course, in the case where there is no spatial dimension, the theory of such stochastic integrals is widely disseminated, but for integrals with respect to space-time white noise (for example) it is far less well-known. It is for this reason that we believe it to be extremely worthwhile making a detailed review of how to give sense to these objects, and moreover to solutions to (1.1) when they exist, in a way that is accessible to practitioners. Although such results are quite well-known in probability theory, the body of literature is very large and generalistic, posing a daunting prospect for a mathematical neuroscientist looking to apply a specific result. The fact that the equation fits into well-studied frameworks also opens up opportunities to apply existing abstract results (for example large deviation principles—see Remark 2.3).

There are in fact two distinct approaches to defining and interpreting the quantity (1.2), both of which allow one to build up a theory of stochastic *partial* differential equations (SPDEs). Although (1.1) does not strictly classify as a SPDE (since there is no derivative with respect to the spatial variable), both approaches provide a rigorous underlying theory upon which to base a study of such equations.

The first approach generalizes the theory of stochastic processes in order to give sense to solutions of SPDEs as random processes that take their values in a Hilbert space of functions [as presented by Da Prato and Zabczyk in (1992) and more recently by Prévôt and Röckner in (2007)]. With this approach, the quantity (1.2) is interpreted as a *Hilbert space-valued* integral i.e. “$\int \mathbf {B}(Y(t))dW(t)$”, where $(Y(t))_{t\ge 0}$ and $(W(t))_{t\ge 0}$ take their values in a Hilbert space of functions, and $\mathbf {B}(Y(t))$ is an operator between Hilbert spaces (depending on $\sigma$). The second approach is that of Walsh [as described in Walsh (1986)], which, in contrast, takes as its starting point a PDE with a random and highly irregular “white-noise” term. This approach develops integration theory with respect to a class of random measures, so that (1.2) can be interpreted as a random field in both $t$ and $x$.

In the theory of SPDEs, there are advantages and disadvantages of taking both approaches. This is also the case with regards to the stochastic neural field Eq. (1.1), as described in the conclusion below (Sect. 5), and it is for this reason that we here review both approaches. Taking the functional approach of Da Prato and Zabczyk is perhaps more straightforward for those with knowledge of stochastic processes, and the existing general results can be applied more directly in order to obtain, for example, existence and uniqueness. This was the path taken in Kuehn and Riedler (2014) where the emphasis was on large deviations, though in a much less general setup than we consider here (see Remark 2.3). However, it can certainly be argued that solutions constructed in this way may be “non-physical”, since the functional theory tends to ignore any spatial regularity properties (solutions are typically $L^2$-valued in the spatial direction). We argue that the approach of Walsh is more suited to looking for “physical” solutions that are at least continuous in the spatial dimension. A comparison of the two approaches in a general setting is presented in Dalang and Quer-Sardanyons (2011) or Jetschke (1982, 1986), and in our setting in Sect. 4 below. Our main conclusion is that in typical cases of interest for practitioners, the approaches are equivalent (see Example 4.2), but one or the other may be more suited to a particular need.

To reiterate, the main aim of this article is to present a review of an existing theory, which is accessible to readers unfamiliar with stochastic partial differential equations, that puts the study of stochastic neural field equations on a rigorous mathematical footing. As a by product we will be able to give general conditions on the functions $G$, $\sigma$ and $w$ that, as far as we know, do not appear anywhere else in the literature and guarantee the existence of a solution to (1.1) in some sense. Moreover, these conditions are weak enough to be satisfied for all typical choices of functions made by practitioners (see Sects. 2.6, 2.7 and 2.8). By collecting all these results in a single place, we hope this will provide a reference for practitioners in future works.

The layout of the article is as follows. We first present in Sect. 2 the necessary material in order to consider the stochastic neural field Eq. (1.1) as an evolution equation in a Hilbert space. This involves introducing the notion of a $Q$-Wiener process taking values in a Hilbert space and stochastic integration with respect to $Q$-Wiener processes. A general existence result from Prato and Zabczyk (1992) is then applied in Sect. 2.5 to yield a unique solution to (1.1) interpreted as a Hilbert space valued process. The second part of the paper switches track, and describes Walsh’s theory of stochastic integration (Sect. 3.1), with a view of giving sense to a solution to (1.1) as a random field in both time and space. To avoid dealing with distribution-valued solutions, we in fact consider a Gaussian noise that is smoothed in the spatial direction (Sect. 3.2), and show that, under some weak conditions, the neural field equation driven by such a smoothed noise has a unique solution in the sense of Walsh that is continuous in both time and space (Sect. 3.3). We finish with a comparison of the two approaches in Sect. 4, and summarize our findings in a conclusion (Sect. 5).

**Notation:** Throughout the article $(\varOmega , {\mathcal {F}} , {\mathbb P} )$ will be a probability space, and $L^2(\varOmega , \mathcal {F}, \mathbb {P})$ will be the space of square-integrable random variables on $(\varOmega , \mathcal {F}, \mathbb {P})$. We will use the standard notation $\mathcal {B}(\mathcal {T})$ to denote the Borel $\sigma$-algebra on $\mathcal {T}$ for any topological space $\mathcal {T}$. The Lebesgue space of $p$-integrable (with respect to the Lebesgue measure) functions over ${\mathbb {R}} ^N$ for $N\in \mathbb {N} = \{1, 2, \dots \}$ will be denoted by $L^p({\mathbb {R}} ^N)$, $p\ge 1$, as usual, while $L^p({\mathbb {R}} ^N, \rho )$, $p\ge 1$, will be the Lebesgue space weighted by a measurable function $\rho :{\mathbb {R}} ^N\rightarrow {\mathbb {R}} ^+$.

## Stochastic neural field equations as evolution equations in Hilbert spaces

As stated in the introduction, the goal of this section is to provide the theory and conditions needed to interpret the solution to (1.1) as a process $(Y(t))_{t\ge 0}$ that takes its values in a Hilbert space of functions i.e. for each $t\ge 0$, $Y(t)$ is a function of the spatial variable $x$. This is in order to try and cast the problem into the well-known theoretical framework of stochastic evolution equations in Hilbert spaces, as detailed in Prato and Zabczyk (1992). In particular we will look for solutions to

2.1 $$\begin{aligned} dY(t) = \left( -Y(t) + \mathbf {F}(Y(t))\right) dt + ``\mathbf {B}(Y(t))dW(t){\text {''}}, \, t\ge 0, \end{aligned}$$

such that $Y(t) \in L^2({\mathbb {R}} ^N, \rho )$ for some measurable $\rho :{\mathbb {R}} ^N\rightarrow {\mathbb {R}} ^+$ (to be determined), where $\mathbf {F}$ is now an operator on $L^2({\mathbb {R}} ^N, \rho )$ given by

$$\begin{aligned} \mathbf {F}(Y(t))(x) = \int _{{\mathbb {R}} ^N}w(x, y)G(Y(t,y))dy, \quad x\in {\mathbb {R}} ^N. \end{aligned}$$

Here $w:{\mathbb {R}} ^N\times {\mathbb {R}} ^N\rightarrow {\mathbb {R}}$ is the neural field kernel, and $G:{\mathbb {R}} \rightarrow {\mathbb {R}}$ is the nonlinear gain function. Note that we have made a slight generalization here in comparison with (1.1) in that we in fact work on ${\mathbb {R}} ^N$, rather than ${\mathbb {R}}$. The term $\mathbf {B}(Y(t))dW(t)$ represents a stochastic differential term that must be made sense of as a differential in the Hilbert space $L^2({\mathbb {R}} ^N, \rho )$. This is done with the help of Sects. 2.1 and 2.2 below.

**Notation:** In this section we will also need the following basic notions from functional analysis. Let $U$ and $H$ be two separable Hilbert spaces. We will write $L_0(U, H)$ to denote the space of all bounded linear operators from $U$ to $H$ with the usual norm^1^ (with the shorthand $L_0(H)$ when $U=H$), and $L_2(U, H)$ for the space of all Hilbert-Schmidt operators from $U$ to $H$, i.e. those bounded linear operators $B:U \rightarrow H$ such that

$$\begin{aligned} \sum _{k\ge 1} \Vert B(e_k)\Vert _{H}^2 <\infty , \end{aligned}$$

for some (and hence all) complete orthonormal systems $\{e_k\}_{k\ge 1}$ of $U$. Finally, a bounded linear operator $Q:U \rightarrow U$ will be said to be trace-class if $\mathrm {Tr}(Q) {:=} \sum _{k\ge 1}\langle Q (e_k), e_k\rangle _U < \infty$, again for some (and hence all) complete orthonormal systems $\{e_k\}_{k\ge 1}$ of $U$.

### Hilbert space valued $Q$-Wiener processes

The purpose of this section is to provide a basic understanding of how we can generalize the idea of an ${\mathbb {R}} ^d$-valued Wiener process to one that takes its values in an infinite dimensional Hilbert space, which for convenience we fix to be $U=L^2({\mathbb {R}} ^N)$ (this is simply for the sake of being concrete).

In the finite dimensional case, it is well-known that ${\mathbb {R}} ^d$-valued Wiener processes are characterized by their $d\times d$ covariance matrices, which are symmetric and non-negative. The basic idea is that in the infinite dimensional setup the covariance matrices are replaced by covariance *operators*, which are linear, non-negative, symmetric and bounded.

Indeed, let $Q:U \rightarrow U$ be a non-negative, symmetric bounded linear operator on $U$. To avoid introducing extra embeddings, we also suppose $\mathrm {Tr}(Q) <\infty$. Then, completely analogously to the finite dimensional case, there exists a sequence of non-negative real numbers $(\lambda _k)_{k\ge 1}$ which are eigenvalues of $Q$, associated with a sequence of eigenfunctions $\{e_k\}_{k\ge 1}$ (i.e. $Qe_k = \lambda _k e_k$) that form a complete orthonormal basis for $U$. Moreover, since $\mathrm {Tr}(Q) <\infty$, it holds that

$$\begin{aligned} \sum _{k=1}^\infty \lambda _k < \infty . \end{aligned}$$

By a $Q$-Wiener process $W = (W(t))_{t\ge 0}$ on $U$ we will simply mean that $W(t)$ can be expanded as

2.2 $$\begin{aligned} W(t) = \sum _{k=1}^\infty \sqrt{\lambda _k}\beta _k(t) e_k, \end{aligned}$$

where $(\beta _k(t))_{t\ge 0}$, $k=1, 2, \dots$ are mutually independent standard real-valued Brownian motions. We note that $W(t)$ exists as a $U$-valued square-integrable random variable i.e. $W(t)\in L^2(\varOmega , \mathcal {F}, \mathbb {P})$.

Equation (2.2) shows the role played by $Q$: the eigenvectors $e_k$ are functions that determine “where” the noise “lives” in $U$, while the eigenvalues $\lambda _k$ determine its dimensionality and relative strength. As an example of a covariance operator^2^, let us compute the covariance operator of $W$. An easy computation based on (2.2) and the elementary properties of the standard real-valued Brownian motion shows that

2.3 $$\begin{aligned} {\mathbb {E}} [\langle W(s),g\rangle _U \langle W(t),h \rangle _U]=(s \wedge t)\langle Q g,h\rangle _U \quad \forall g,\,h\in U. \end{aligned}$$

It turns out that $W$ is white in both space and time. The whiteness in time is apparent from the above expression. The whiteness in space is shown explicitly in Sect. 2.7.

### Stochastic integration with respect to $Q$-Wiener processes

The second point is that we would like to be able to define stochastic integration with respect to these Hilbert space valued Wiener processes. In particular we must determine for which integrands this can be done [exactly as in Prato and Zabczyk (1992)].

As above, let $U = L^2({\mathbb {R}} ^N)$, $Q:U \rightarrow U$ a non-negative, symmetric bounded linear operator on $U$ such that $\mathrm {Tr}(Q) <\infty$, and $W = (W(t))_{t\ge 0}$ be a $Q$-Wiener process on $U$ [given by (2.2)].

Unfortunately, in order to define stochastic integrals with respect to $W$, we need a couple of technical definitions from functional analysis. This is simply in order to control the convergence of the infinite series that appear in the construction, as we will see in the example below. Indeed, let $Q^\frac{1}{2}(U)$ be the subspace of $U$, which is a Hilbert space under the inner product

$$\begin{aligned} \langle u, v \rangle _{Q^\frac{1}{2}(U)} {:=} \left\langle Q^{-\frac{1}{2}}u, Q^{-\frac{1}{2}}v \right\rangle _{U}, \quad u, v \in Q^{\frac{1}{2}}(U). \end{aligned}$$

$Q^\frac{1}{2}(U)$ is in fact simply the space generated by the orthonormal basis $\{\sqrt{\lambda _k}e_k\}$ whenever $\{e_k\}$ is the orthonormal basis for $U$ consisting of eigenfunctions of $Q$. Moreover, let $H = L^2({\mathbb {R}} ^N, \rho )$ for some measurable $\rho :{\mathbb {R}} ^N\rightarrow {\mathbb {R}} ^+$ (again this is just for the sake of concreteness—one could instead take any separable Hilbert space). It turns out that the space $L_2(Q^\frac{1}{2}(U), H)$ of all Hilbert-Schmidt operators from $Q^\frac{1}{2}(U)$ into $H$ plays an important role in the theory of stochastic integration with respect to $W$, and for this reason we detail the following simple but illuminating example.

#### *Example 2.1*

Let $B: U \rightarrow H$ be a bounded linear operator from $U$ to $H$ i.e. $B\in L_0(U, H)$. Then, by definition,

$$\begin{aligned} \Vert B\Vert ^2_{L_2(Q^\frac{1}{2}(U), H)}&= \sum _{k=1}^\infty \Vert B (Q^\frac{1}{2}(e_k))\Vert ^2_{H}\\&\le \Vert B\Vert ^2_{L_0(U, H)}\sum _{k=1}^\infty \Vert Q^\frac{1}{2}(e_k)\Vert ^2_{U}\\&= \Vert B\Vert ^2_{L_0(U, H)}\sum _{k=1}^\infty \langle Q^\frac{1}{2}(e_k), Q^\frac{1}{2}(e_k) \rangle _U\\&= \Vert B\Vert ^2_{L_0(U, H)}\sum _{k=1}^\infty \langle Q(e_k), e_k \rangle _U = \Vert B\Vert ^2_{L_0(U, H)}\mathrm{Tr}(Q) <\infty , \end{aligned}$$

since $\mathrm {Tr}(Q)<\infty$, where $\{e_k\}_{k\ge 1}$ is again a complete orthonormal system for $U$. In other words $B\in L_0(U, H) \Rightarrow B\in L_2(Q^\frac{1}{2}(U), H)$.

The main point of the section is the following. According to the construction detailed in Chapter 4 of Prato and Zabczyk (1992), we have that for a (random) process $(\varPhi (t))_{t\ge 0}$ the integral

2.4 $$\begin{aligned} \int _0^t\varPhi (s)dW(s) \end{aligned}$$

has a sense as an element of $H$ when $\varPhi (s)\in L_2(Q^\frac{1}{2}(U), H)$, $\varPhi (s)$ is knowable^3^ at time $s$, and if

$$\begin{aligned} \mathbb {P}\left( \int _0^t \Vert \varPhi (s)\Vert ^2_{L_2(Q^\frac{1}{2}(U), H)} ds < \infty \right) = 1. \end{aligned}$$

Now in view of Example 2.1, the take-away message is simply that the stochastic integral (2.4) has a sense in $H$ if $\varPhi (s): U \rightarrow H$ is a bounded linear operator i.e. is in $L_0(U, H)$ for all $s\in [0,t]$, and that the norm of $\varPhi (s)$ is bounded on $[0,t]$. In fact this is the only knowledge that will be needed below.

### The stochastic neural field equation: interpretation in language of Hilbert space valued processes

With the previous two sections in place, we can now return to (2.1) and interpret it (and in particular the noise term) in a rigorous way. Indeed, as above, let $W$ be an $L^2({\mathbb {R}} ^N)$-valued $Q$-Wiener process, with $Q$ a non-negative, symmetric bounded linear operator on $L^2({\mathbb {R}} ^N)$ such that $\mathrm {Tr}(Q) <\infty$ (trace-class). The rigorous interpretation of (2.1) as an equation for a process $(Y(t))_{t\ge 0}$ taking its values in the Hilbert space $L^2({\mathbb {R}} ^N, \rho )$ is then

2.5 $$\begin{aligned} dY(t) = \left( -Y(t) + \mathbf {F}(Y(t))\right) dt + \mathbf {B}(Y(t))dW(t), \quad Y(0) = Y_0\in L^2({\mathbb {R}} ^N, \rho )\qquad \end{aligned}$$

where $\mathbf {B}$ is a map from $L^2({\mathbb {R}} ^N, \rho )$ into the space of bounded linear operators $L_0(L^2({\mathbb {R}} ^N), L^2({\mathbb {R}} ^N, \rho ))$. Note that if $\mathbf {B}$ is such a map, then the integrated noise term of this equation has a sense thanks to Sect. 2.2.

We in fact work with a general map $\mathbf {B}$ satisfying a Lipschitz condition (see below), but we keep in mind the following example which provides the link with the diffusion coefficient $\sigma$ in (1.1):

2.6 $$\begin{aligned} \mathbf {B}(h)(u)(x) = \sigma (h(x))\int _{{\mathbb {R}} ^N}\varphi (x-y)u(y)dy,\quad x\in {\mathbb {R}} ^N, \end{aligned}$$

for $h\in L^2({\mathbb {R}} ^N, \rho )$ and $u\in L^2({\mathbb {R}} ^N)$, where $\sigma$ and $\varphi$ are some functions that must be chosen to ensure the conditions stated below are satisfied. We detail potential choices of $\sigma$ and $\varphi$ (and their significance from a modeling point of view—in particular how $\varphi$ controls the spatial correlation) in Sect. 2.7 below.

To summarize, we are here concerned with the solvability of (2.5) in $L^2({\mathbb {R}} ^N, \rho )$ (for some measurable $\rho :{\mathbb {R}} ^N \rightarrow {\mathbb {R}} ^+$ to be determined), where

2.7 $$\begin{aligned} \mathbf {F}(h)(x) = \int _{{\mathbb {R}} ^N}w(x, y)G(h(y))dy, \quad x\in {\mathbb {R}} ^N,\ h\in L^2({\mathbb {R}} ^N, \rho ), \end{aligned}$$

and $\mathbf {B}: L^2({\mathbb {R}} ^N, \rho )\rightarrow L_0(L^2({\mathbb {R}} ^N), L^2({\mathbb {R}} ^N,\rho ))$. To this end, we make the following two Lipschitz assumptions on $\mathbf {B}$ and the nonlinear gain function $G$:

- $\mathbf {B}: H \rightarrow L_0( U, H)$ is such that
  $$\begin{aligned} \Vert \mathbf {B}(g) - \mathbf {B}(h)\Vert _{L_0(U, H)} \le C_\sigma \Vert g-h\Vert _{U}, \quad g, h \in L^2({\mathbb {R}} ^N, \rho ), \end{aligned}$$
  where $U= L^2({\mathbb {R}} ^N)$ and $H=L^2({\mathbb {R}} ^N, \rho )$ for notational simplicity;
- $G:{\mathbb {R}} \rightarrow {\mathbb {R}}$ is bounded and globally Lipschitz i.e such that there exists a constant $C_G$ with $\sup _{a\in {\mathbb {R}} }|G(a)| \le C_G$ and
  $$\begin{aligned} |G(a) - G(b)| \le C_G|a- b|, \qquad \forall a, b \in {\mathbb {R}} . \end{aligned}$$
  Typically the nonlinear gain function $G$ is taken to be a sigmoid function, for example $G(a) = (1+e^{-a})^{-1}$, $a\in {\mathbb {R}}$, which certainly satisfies this assumption.

### Discussion of conditions on the neural field kernel $w$ and $\rho$

Of particular interest to us are the conditions on the neural field kernel $w$ which will allow us to prove existence and uniqueness of a solution to (2.5) by quoting a standard result from Prato and Zabczyk (1992).

In Kuehn and Riedler (2014, footnote 1) it is suggested that the condition

$$\begin{aligned} \int _{{\mathbb {R}} ^N}\int _{{\mathbb {R}} ^N} |w(x, y)|^2 dx dy <\infty \quad (\mathbf C1 ) \end{aligned}$$

together with symmetry of $w$ is enough to ensure that there exists a unique $L^2({\mathbb {R}} ^N)$-valued solution to (2.5). However, the problem is that it does not follow from (**C1**) that the operator $\mathbf {F}$ is stable on the space $L^2({\mathbb {R}} ^N)$. For instance, suppose that in fact $G \equiv 1$ (so that $G$ is trivially globally Lipschitz). Then for $h\in L^2({\mathbb {R}} ^N)$ (and assuming $w\ge 0$) we have that

2.8 $$\begin{aligned} \Vert \mathbf F (h)\Vert ^2_{L^2({\mathbb {R}} ^N)} = \int _{{\mathbb {R}} ^N}\Vert w(x, \cdot )\Vert _{L^1({\mathbb {R}} ^N)}^2dx. \end{aligned}$$

The point is that we can choose positive $w$ such that (**C1**) holds, while (2.8) is not finite. For example in the case $N=1$ we could take $w(x, y) = (1+|x|)^{-1}(1+|y|)^{-1}$ for $x, y \in {\mathbb {R}}$. In such a case the Eq. (2.5) is ill-posed: if $Y(t)\in L^2({\mathbb {R}} )$ then $F(t, Y(t))$ is not guaranteed to be in $L^2({\mathbb {R}} )$, which in turn implies that $Y(t)\not \in L^2({\mathbb {R}} )$!

With this in mind we argue two points. Firstly, if we want a solution in $L^2({{\mathbb {R}} ^N})$, we must make the additional strong assumption that

$$\begin{aligned} \forall x\in {{\mathbb {R}} ^N}\ (y\mapsto w(x, y))\in L^1({{\mathbb {R}} ^N}), \quad \mathrm {and}\quad \Vert w(x, \cdot )\Vert _{L^1({{\mathbb {R}} ^N})} \in L^2({{\mathbb {R}} ^N}). \quad (\mathbf C2 ) \end{aligned}$$

Indeed, below we will show that $\mathbf (C1)$ together with $\mathbf (C2)$ are enough to yield the existence of a unique $L^2({{\mathbb {R}} ^N})$-valued solution to (2.5).

On the other hand, if we don’t want to make the strong assumptions that (**C1**) and (**C2**) hold, then we have to work instead in a weighted space $L^2({{\mathbb {R}} ^N}, \rho )$, in order to ensure that $\mathbf {F}$ is stable. In this case, we will see that if

$$\begin{aligned} \exists \ \rho _w\in L^1({{\mathbb {R}} ^N})\quad \mathrm {s.t.}\quad \int _{{\mathbb {R}} ^N} |w(x, y)|\rho _w(x)dx \le \Lambda _w\rho _w(y)\ \forall y\in {{\mathbb {R}} ^N}, \quad (\mathbf C1' ) \end{aligned}$$

for some $\Lambda _w>0$, and

$$\begin{aligned} \forall x\in {{\mathbb {R}} ^N}\ (y\mapsto w(x, y))\in L^1({{\mathbb {R}} ^N}), \mathrm {and}\ \sup _{x\in {{\mathbb {R}} ^N}}\Vert w(x,\cdot )\Vert _{L^1({{\mathbb {R}} ^N})} \le C_w \quad (\mathbf C2' ) \end{aligned}$$

for some constant $C_w$, then we can prove the existence of a unique $L^2({{\mathbb {R}} ^N}, \rho _w)$-valued solution to (2.5).

Condition $(\mathbf C1' )$ is in fact a non-trivial eigenvalue problem, and it is not straightforward to see whether it is satisfied for a given function $w$. However, we chose to state the theorem below in a general way, and then below provide some important examples of when it can be applied.

We will discuss these abstract conditions from a modeling point of view below. However, we first present the existence and uniqueness result.

### Existence and uniqueness

#### **Theorem 2.2**

Suppose that the neural field kernel $w$ either

(i) satisfies conditions (**C1**) and (**C2**); or
(ii) satisfies conditions (**C1’**) and (**C2’**).

If (i) holds set $\rho _w\equiv 1$, while if (ii) holds let $\rho _w$ be the function appearing in condition (**C1’**).

Then, whenever $Y_0$ is an $L^2({{\mathbb {R}} ^N}, \rho _w)$-valued random variable with finite $p$-moments for all $p\ge 2$, the neural field Eq. (2.5) has a unique solution taking values in the space $L^2({{\mathbb {R}} ^N}, \rho _w)$. To be precise, there exists a unique $L^2({{\mathbb {R}} ^N}, \rho _w)$-valued process $(Y(t))_{t\ge 0}$ such that for all $T>0$

$$\begin{aligned} \mathbb {P}\left( \int _0^T \Vert Y(s)\Vert ^2_{L^2({{\mathbb {R}} ^N}, \rho _w)}ds < \infty \right) = 1, \end{aligned}$$

and

$$\begin{aligned} Y(t) = e^{-t}Y_0 + \int _0^te^{-(t-s)}\mathbf {F}(Y(s))ds +\int _0^te^{-(t-s)} \mathbf {B}(Y(s))dW(s), \quad \mathbb {P}-a.s. \end{aligned}$$

Moreover, $(Y(t))_{t\ge 0}$ has a continuous modification, and satisfies the bounds

2.9 $$\begin{aligned} \sup _{t\in [0, T]} \mathbb {E}\left[ \, \Vert Y(t)\Vert _{L^2({\mathbb {R}} ^N, \rho _w)}^p \,\right] \le C^{( p)}_{T}\left( 1 + \mathbb {E}\left[ \, \Vert Y_0\Vert ^p_{L^2({\mathbb {R}} ^N, \rho _w)} \,\right] \right) , \quad T>0,\quad \end{aligned}$$

for all $p\ge 2$, while for $p>2$,

2.10 $$\begin{aligned} \mathbb {E}\left[ \, \sup _{t\in [0, T]}\Vert Y(t)\Vert _{L^2({\mathbb {R}} ^N, \rho _w)}^p \,\right] \le C^{(p )}_T\left( 1 + \mathbb {E}\left[ \, \Vert Y_0\Vert ^p_{L^2({\mathbb {R}} ^N, \rho _w)} \,\right] \right) \quad T>0.\qquad \end{aligned}$$

#### *Proof*

We simply check the hypotheses of Prato and Zabczyk (1992, Theorem 7.4) (a standard reference in the theory) in both cases (i) and (ii). This involves showing that (a) $\mathbf {F}: L^2({{\mathbb {R}} ^N},\rho _w) \rightarrow L^2({{\mathbb {R}} ^N},\rho _w)$; (b) the operator $\mathbf {B}(h)\in L_2(Q^\frac{1}{2}(U), H)$, for all $h\in H$ [recalling that $U=L^2({\mathbb {R}} ^N)$ and $H=L^2({\mathbb {R}} ^N, \rho )$]; and (c) $\mathbf {F}$ and $\mathbf {B}$ are globally Lipschitz.

(a): We check that the function $\mathbf {F}: L^2({{\mathbb {R}} ^N},\rho _w) \rightarrow L^2({{\mathbb {R}} ^N},\rho _w)$. In case (i) this holds since $\rho _w\equiv 1$ and for any $h\in L^2({{\mathbb {R}} ^N})$

$$\begin{aligned} \Vert \mathbf {F}(h)\Vert ^2_{ L^2({{\mathbb {R}} ^N})}&= \int _{{{\mathbb {R}} ^N}}\left| \,\,\int _{{{\mathbb {R}} ^N}}w(x,y)G(h(y))dy\right| ^2dx\\&\le C_G^2 \int _{{{\mathbb {R}} ^N}}\Vert w(x, \cdot )\Vert _{L^1({{\mathbb {R}} ^N})}^2dx < \infty , \end{aligned}$$

by assumption (**C2**). Similarly in case (ii) for any $h\in L^2({{\mathbb {R}} ^N}, \rho _w)$

$$\begin{aligned} \Vert \mathbf {F}(h)\Vert ^2_{ L^2({{\mathbb {R}} ^N}, \rho _w)}&= \int _{{{\mathbb {R}} ^N}}\left| \,\,\int _{{{\mathbb {R}} ^N}}w(x,y)G(h(y))dy\right| ^2 \rho _w(x)dx\\&\le C_G^2 \sup _{x\in {{\mathbb {R}} ^N}}\Vert w(x, \cdot )\Vert _{L^1({{\mathbb {R}} ^N})}^2\Vert \rho _w\Vert _{L^1({{\mathbb {R}} ^N})} < \infty . \end{aligned}$$

Hence in either case $\mathbf {F}$ in fact maps $L^2({{\mathbb {R}} ^N}, \rho _w)$ into a metric ball in $L^2({{\mathbb {R}} ^N}, \rho _w)$.

(b): To show (b) in both cases, we know by Example 2.1 that for $h\in H$, $\mathbf {B}(h)\in L_2(Q^\frac{1}{2}(U), H)$ whenever $\mathbf {B}(h)\in L_0(U, H)$, which is true by assumption.

(c): To show (c), we first want $\mathbf {F}: L^2({{\mathbb {R}} ^N}, \rho _w) \rightarrow L^2({{\mathbb {R}} ^N}, \rho _w)$ to be globally Lipschitz. To this end, for any $g, h\in L^2({{\mathbb {R}} ^N}, \rho _w)$, we see that in either case

$$\begin{aligned}&\Vert \mathbf {F}(g) - \mathbf {F}(h)\Vert _{L^2({{\mathbb {R}} ^N}, \rho _w)}^2 = \int _{{{\mathbb {R}} ^N}}|\mathbf {F}(g) - \mathbf {F}(h)|^2(x)\rho _w(x)dx\\&\qquad \le \int _{{\mathbb {R}} ^N}\left( \,\,\int _{{\mathbb {R}} ^N}|w(x,y)|\left| G(g(y)) - G(h(y))\right| dy \right) ^2\rho _w(x)dx\\&\qquad \le C^2_G \int _{{\mathbb {R}} ^N}\left( \,\,\int _{{\mathbb {R}} ^N}|w(x,y)|\left| g(y) - h(y)\right| dy \right) ^2\rho _w(x)dx, \end{aligned}$$

where we have used the Lipschitz property of $G$. Now in case (i) it clearly follows from the Cauchy–Schwartz inequality that

$$\begin{aligned} \Vert \mathbf {F}(g) - \mathbf {F}(h)\Vert _{L^2({{\mathbb {R}} ^N})}^2 \le C^2_G \left( \,\,\int _{{\mathbb {R}} ^N}\int _{{\mathbb {R}} ^N} |w(x, y)|^2 dx dy \right) \left\| g - h\right\| _{L^2({{\mathbb {R}} ^N})}\!, \end{aligned}$$

so that by condition (**C1**), $\mathbf {F}$ is indeed Lipschitz.

In case (ii), by Cauchy–Schwartz and the specific property of $\rho _w$ given by (**C1’**), we see that

$$\begin{aligned}&\Vert \mathbf {F}(g) - \mathbf {F}(h)\Vert _{L^2({{\mathbb {R}} ^N}, \rho _w)}^2 \\&\qquad \le C^2_G \sup _{x\in {{\mathbb {R}} ^N}}\Vert w(x, \cdot )\Vert _{L^1({{\mathbb {R}} ^N})} \int _{{\mathbb {R}} ^N} \left| g(y) - h(y)\right| ^2\left( \,\,\int _{{\mathbb {R}} ^N} |w(x,y)| \rho _w(x)dx\right) dy\\&\qquad \le C^2_G\Lambda _w \sup _{x\in {{\mathbb {R}} ^N}}\Vert w(x, \cdot )\Vert _{L^1({{\mathbb {R}} ^N})} \Vert g - h\Vert _{L^2({{\mathbb {R}} ^N}, \rho _w)}^2, \end{aligned}$$

so that again $\mathbf {F}$ is Lipschitz. Since we have assumed that $\mathbf {B}:H\rightarrow L_0(U,H)$ is Lipschitz, we are done.$\square$

#### *Remark 2.3*

(Large Deviation Principle) The main focus of Kuehn and Riedler (2014) was a large deviation principle for the stochastic neural field Eq. (2.5) with small noise, but in a less general situation than we consider here. In particular, the authors only considered the neural field equation driven by a simple additive noise, white in both space and time.

We would therefore like to remark that in our more general case, and under much weaker conditions than those imposed in Kuehn and Riedler (2014) (our conditions are for example satisfied for a connectivity function $w$ that is homogeneous, as we will see in Example 2 below), an LDP result for the solution identified by the above theorem still holds and can be quoted from the literature. Indeed, such a result is presented in Peszat (1994, Theorem 7.1). The main conditions required for the application of this result have essentially already been checked above (global Lipschitz properties of $\mathbf {F}$ and $\mathbf {B}$), and it thus remains to check conditions (E.1)–(E.4) as they appear in Peszat (1994). In fact these are trivialities, since the strongly continuous contraction semigroup $S(t)$ is generated by the identity in our case.

### Discussion of conditions on $w$ and $\rho$ in practice

Our knowledge about the kinds of neural field kernels that are found in the brains of mammals is still quite limited. Since visual perception is the most active area of research, it should not come as a surprise that it is in cortical regions involved in visual perception that this knowledge is the most extensive, and in particular in the primary visual area called V1 in humans. In models of this region it is usually assumed that $w$ is the sum of two parts: a local part $w_{loc}$ corresponding to local neuronal connections, and a non-local part $w_{lr}$ corresponding to longer range connections. As suggested in Lund et al. (2003), Mariño et al. (2005), $w_{loc}$ is well approximated by a Gaussian function (or a difference of of such functions, see below):

2.11 $$\begin{aligned} w_{loc}(x, y)=K \exp (-|x - y|^2/2\beta _{loc}^2) \quad x, y \in {\mathbb {R}} ^N,\,K > 0, \end{aligned}$$

where $\beta _{loc}$ is the extent of the local connectivity. Hence $w_{loc}$ is isotropic and homogeneous. In fact for practitioners, a very common assumption on $w$ is that it is homogeneous and in $L^1({{\mathbb {R}} ^N})$, which thus concentrates on modeling the local interactions (Bressloff and Folias 2004; Bressloff and Webber 2012; Bressloff and Wilkerson 2012; Folias and Bressloff 2004; Kilpatrick and Ermentrout 2013; Owen et al. 2007). However, when $w$ is homogeneous it is clear that neither (**C1**) nor (**C2**) of the above theorem are satisfied, and so we instead must try to show that (**C1’**) is satisfied [(**C2’**) trivially holds], and look for solutions in a weighted $L^2$ space. This is done in the second example below.

Long range connectivity is best described by assuming $N=2$. It is built upon the existence of maps of orientation sensitivity in which the preferred visual orientation at each point $x$ is represented by a function $\theta (x) \in [0,\pi )$. This function is smooth except at countably many points called the pinwheels where it is undefined^4^. Depending on the species, the long range connections feature an anisotropy, meaning that they tend to align themselves with the preferred orientation at $x$. On way to take this into account is to introduce the function $A(\chi ,x)=\exp [-((1-\chi )^2x_1^2+x_2^2)/2\beta _{lr}^2]$, where $x=(x_1,x_2)$, $\chi \in [0,1)$, and $\beta _{lr}$ is the extent of the long range connectivity. When $\chi =0$ there is no isotropy (as for the macaque monkey for example) and when $\chi \in (0,1)$ there is some anisotropy (as for the tree shrew, for example). Let $R_\alpha$ represent the rotation by angle $\alpha$ around the origin. The long range neural field kernel is then defined by (Baker and Cowan 2009; Bressloff 2003)

$$\begin{aligned} w_{lr}(x,y)=\varepsilon _{lr}A(\chi ,R_{-2\theta (x)}(x-y)) \cdot G_{\beta _\theta }(\theta (x)-\theta (y)), \end{aligned}$$

where $\varepsilon _{lr} \ll 1$ and $G_{\beta _\theta }$ is the one-dimensional Gaussian density with 0 mean and variance $\beta _\theta ^2$. Note that $w_{lr}$ is not homogeneous, even in the case $\chi =0$, because $\theta (x)-\theta (y)$ is not a function of $x-y$. It is easy to verify that $w_{lr} \in L^2({\mathbb {R}} ^2)$.

Combining the local and non-local parts, one then writes for the neural field kernel of the primary visual area:

2.12 $$\begin{aligned} w_{pva}(x,y)=w_{loc}(x-y)+w_{lr}(x,y). \end{aligned}$$

In view of our results, in the case where $w = w_{pva}$, since the first part is homogeneous while the second is non-homogeneous but is in $L^2({\mathbb {R}} ^2)$, we need a combination of the results above. Indeed, the homogeneous part dictates to work in $L^2({\mathbb {R}} ^2,\rho _{w_{loc}})$ ($\rho _{w_{loc}} \in L^1({{\mathbb {R}} ^2})$). The second kernel dictates to work in $L^2({\mathbb {R}} ^2)$. But $L^2({\mathbb {R}} ^2) \subset L^2({\mathbb {R}} ^2,\rho _{w_{loc}})$, because, as shown in Example 2 below $\rho _{w_{loc}}$ can be chosen to be bounded, and hence there is no problem.

Another commonly used type of (homogeneous) neural field kernel, when modeling excitatory and inhibitory populations of neurons is the so-called “Mexican hat” kernel defined by

2.13 $$\begin{aligned} w_{mh}(x, y)\!=\!K_1 \exp (-|x\!-\!y|^2/2\beta _1^2)\!-\!K_2 \exp (-|x\!-\!y|^2/2\beta _2^2),\, x, y \in {\mathbb {R}} ^N,\nonumber \\ \end{aligned}$$

for some $K_1,\,K_2 >0$. If $\beta _2 > \beta _1$ and $K_1 > K_2$ for example, this is locally excitatory and remotely inhibitory.

It is also important to mention the role of $\rho _w$ from a modeling perspective. The first point is that in the case where $w$ is homogeneous, it is very natural to look for solutions that live in $L^2({\mathbb {R}} ^N, \rho )$ for some $\rho \in L^1({{\mathbb {R}} ^N})$, rather than in $L^2({\mathbb {R}} ^N)$. This is because in the deterministic case (see Ermentrout and McLeod 1993), solutions of interest are of the form of traveling waves, which are constant at $\infty$, and thus are not integrable.

Moreover, we emphasize that in Theorem 2.2 and the examples in the next section we identify a single $\rho _w\in L^1({{\mathbb {R}} ^N})$ so that the standard existence result of Prato and Zabczyk (1992) can be directly applied through Theorem 2.2. We do not claim that this is the only weight $\rho$ for which the solution can be shown to exist in $L^2({\mathbb {R}} ^N, \rho )$ (see also Example 2 below).

#### *Remark 2.4*

If we replace the spatial coordinate space ${{\mathbb {R}} ^N}$ by a bounded domain $\mathcal {D}\subset {{\mathbb {R}} ^N}$, so that the neural field Eq. (2.5) describes the activity of a neuron found at position $x\in \mathcal {D}$ then checking the conditions as done Theorem 2.2 becomes rather trivial (under appropriate boundary conditions). Indeed, by doing this one can see that there exists a unique $L^2(\mathcal {D})$-valued solution to (2.5) under the condition (**C2’**) only (with ${{\mathbb {R}} ^N}$ replaced by $\mathcal {D}$). Although working in a bounded domain seems more physical (since any physical section of cortex is clearly bounded), the unbounded case is still often used, see Bressloff and Webber (2012) or the review Bressloff (2012), and is mathematically more interesting. The problem in passing to the unbounded case stems from the fact that the nonlocal term in (2.5) naturally ‘lives’ in the space of bounded functions, while according to the theory the noise naturally lives in an $L^2$ space. These are not compatible when the underlying space is unbounded.

### Discussion of the noise term in (2.5)

It is important to understand the properties of the noise term in the neural field Eq. (2.5) which we now know has a solution in some sense. As mentioned above, one particular form of the noise operator $\mathbf {B}$ that is of special importance from a modeling point of view is given by (2.6) i.e.

2.14 $$\begin{aligned} \mathbf {B}(h)(u)(x) = \sigma (h(x))\int _{{\mathbb {R}} ^N}\varphi (x-y)u(y)dy,\quad x\in {\mathbb {R}} ^N, \end{aligned}$$

for $h\in L^2({\mathbb {R}} ^N, \rho )$ and $u\in L^2({\mathbb {R}} ^N)$, and some functions $\sigma$, and $\varphi$. This is because such noise terms are spatially correlated depending on $\varphi$ (as we will see below) and make the link with the original Eq. (1.1) considered in Bressloff and Webber (2012), where spatial correlations are important.

An obvious question is then for which choices of $\sigma$ and $\varphi$ can we apply the above results? In particular we need to check that $\mathbf {B}(h)$ is a bounded linear operator from $L^2({\mathbb {R}} ^N)$ to $L^2({\mathbb {R}} ^N, \rho )$ for all $h\in L^2({\mathbb {R}} ^N, \rho )$, and that $\mathbf {B}$ is Lipschitz (assuming as usual that $\rho \in L^1({\mathbb {R}} ^N)$).

To this end, suppose $\varphi \in L^2({\mathbb {R}} ^N)$ and that there exists a constant $C_\sigma$ such that

2.15 $$\begin{aligned} |\sigma (a) - \sigma (b)| \le C_\sigma |a-b|,\, \, \mathrm {and}\, \, |\sigma (a)|\le C_\sigma (1+|a|), \quad \forall a, b\in {\mathbb {R}} . \end{aligned}$$

In other words $\sigma :{\mathbb {R}} \rightarrow {\mathbb {R}}$ is assumed to be Lipschitz and of linear growth. Then for any $h\in L^2({\mathbb {R}} ^N, \rho )$ and $u\in L^2({\mathbb {R}} ^N)$,

$$\begin{aligned} \Vert \mathbf {B}(h)(u)\Vert ^2_{L^2({\mathbb {R}} ^N, \rho )}&= \int _{{\mathbb {R}} ^N}\sigma ^2(h(x))\left( \,\,\int _{{\mathbb {R}} ^N}\varphi (x-y)u(y)dy\right) ^2\rho (x)dx\\&\le 2\Vert u\Vert ^2_{L^2({\mathbb {R}} ^N)}\Vert \varphi \Vert ^2_{L^2({\mathbb {R}} ^N)}(\Vert \rho \Vert _{L^1({\mathbb {R}} ^N)} + \Vert h\Vert ^2_{L^2({\mathbb {R}} ^N, \rho )}). \end{aligned}$$

Thus $\mathbf {B}(h)$ is indeed a bounded linear operator from $L^2({\mathbb {R}} ^N)$ to $L^2({\mathbb {R}} ^N, \rho )$. Moreover, a similar calculation yields the Lipschitz property of $\mathbf {B}$, so that the above results can be applied. In particular our results hold when $\sigma (a) = \lambda a$, for some $\lambda \in {\mathbb {R}}$. This is important because it is this choice of $\sigma$ that is used for the simulations carried out in Bressloff and Webber (2012, Section 2.3).

To see the spatial correlation in the noise term in (2.5) when $\mathbf {B}$ has the form (2.14) with $\varphi \in L^2({\mathbb {R}} ^N)$, consider the case $\sigma \equiv 1$ (so that the noise is purely additive). Then

$$\begin{aligned} \int _0^t\mathbf {B}(Y(t))dW(t) = \int _0^tBdW(t) {=:} X(t), \quad t\ge 0, \end{aligned}$$

where

$$\begin{aligned} B(u)(x) = \int _{{{\mathbb {R}} ^N}} \varphi (x-y)u(y)dy, \quad x\in {{\mathbb {R}} ^N},\ u\in L^2({{\mathbb {R}} ^N}), \end{aligned}$$

and $X(t)$ is a well-defined $L^2({{\mathbb {R}} ^N}, \rho )$-valued process since $B$ is bounded from $L^2({{\mathbb {R}} ^N})$ into $L^2({{\mathbb {R}} ^N}, \rho )$ (see Sect. 2.2). Moreover, by Theorem 5.2^5^ of Prato and Zabczyk (1992), $(X(t))_{t\ge 0}$ is Gaussian with mean zero and

$$\begin{aligned} \mathrm {Cov} \left( X(t)X(s)\right) = s\wedge t BQB^*, \quad s, t \ge 0, \end{aligned}$$

where $B^*:L^2({{\mathbb {R}} ^N}, \rho )\rightarrow L^2({{\mathbb {R}} ^N})$ is the adjoint of $B$. In other words, for all $g, h \in L^2({{\mathbb {R}} ^N}, \rho )$, $s, t\ge 0$, we have, by definition of the covariance operator, that

$$\begin{aligned} \mathbb {E}\left[ \, \langle g, X(s) \rangle _{L^2({{\mathbb {R}} ^N}, \rho )}\langle h, X(t) \rangle _{L^2({{\mathbb {R}} ^N}, \rho )} \,\right] = s\wedge t\langle BQB^*g, h\rangle _{L^2({{\mathbb {R}} ^N}, \rho )}. \end{aligned}$$

That is, for any $g, h \in L^2({{\mathbb {R}} ^N}, \rho )$

2.16 $$\begin{aligned}&\int _{{\mathbb {R}} ^N}\int _{{\mathbb {R}} ^N}\mathbb {E}\left[ \, X(s, x) X(t, y) \,\right] g(x)h(y)\rho (x)\rho (y)dxdy = s\wedge t\left\langle QB^*h, B^*g\right\rangle _{L^2({{\mathbb {R}} ^N})}\nonumber \\&\qquad \qquad \qquad = s\wedge t\int _{{\mathbb {R}} ^N} QB^*g(z)B^*h(z)dz\nonumber \\&.\qquad \qquad \qquad = s\wedge t\int _{{\mathbb {R}} ^N} Q^{1/2}B^*g(z)Q^{1/2}B^*h(z)dz. \end{aligned}$$

Now, by definition, for $u\in L^2({{\mathbb {R}} ^N})$ and $f\in L^2({{\mathbb {R}} ^N}, \rho )$

$$\begin{aligned} \int _{{\mathbb {R}} ^N} u(y) B^*(f)(y)dy&= \int _{{\mathbb {R}} ^N} B(u)(x)f(x)\rho (x)dx \\&= \int _{{\mathbb {R}} ^N} u(y)\int _{{\mathbb {R}} ^N} \varphi (x-y)f(x)\rho (x)dxdy \end{aligned}$$

so that $B^*(f)(y) = \int _{{\mathbb {R}} ^N}\varphi (x-y)f(x)\rho (x)dx$. Using this in (2.16), we see that

$$\begin{aligned}&\int _{{\mathbb {R}} ^N}\int _{{\mathbb {R}} ^N}\mathbb {E}\left[ \, X(s, x) X(t, y) \,\right] g(x)h(y)\rho (x)\rho (y)dxdy\\&\quad = s\wedge t\int _{{\mathbb {R}} ^N} \left( \,\,\int _{{\mathbb {R}} ^N} Q^\frac{1}{2}\varphi (x-z)g(x)\rho (x)dx\right) \left( \,\,\int _{{{\mathbb {R}} ^N}}Q^\frac{1}{2}\varphi (y-z)h(y)\rho (y)dy\right) dz, \end{aligned}$$

for all $g, h \in L^2({{\mathbb {R}} ^N}, \rho )$, since $Q$ is a linear operator and is self-adjoint. We can then conclude that

2.17 $$\begin{aligned} \mathbb {E}\left[ \, X(s, x) X(t, y) \,\right] \!=\!s\wedge t\int _{{\mathbb {R}} ^N} Q^\frac{1}{2}\varphi (x\!-\!z)Q^\frac{1}{2}\varphi (y\!-\!z)dz \!=\! (s\wedge t)c(x-y),\qquad \quad \end{aligned}$$

where $c(x) = Q^\frac{1}{2}\varphi *Q^\frac{1}{2}\widetilde{\varphi }(x)$ and $\widetilde{\varphi }(x)=\varphi (-x)$. Hence $(X(t))_{t\ge 0}$ is white in time but stationary and colored in space with covariance function $(s\wedge t)c(x)$. We remark that the manipulations above are certainly not new [they are for example used in Brzeźniak and Peszat (1999)], but they illustrate nicely the spatial correlation property of the noise we consider.

We conclude that (2.14) is exactly the rigorous interpretation of the noise described in Bressloff and Webber (2012), when interpreting a solution to the stochastic neural field equation as a process taking values in $L^2({{\mathbb {R}} ^N}, \rho _w)$.

#### *Remark 2.5*

Note that in the case where $B$ is the identity, $X(t)=W(t)$. We can, at least formally, carry out the above computation with $\varphi =\delta _0$ and find that

2.18 $$\begin{aligned} \mathbb {E}\left[ \, W(s,x)W(t,y) \,\right] =(s\wedge t) Q \delta _0(x-y), \end{aligned}$$

which yields for any $g,\,h \in L^2({\mathbb {R}} ^N)$

$$\begin{aligned}&\mathbb {E}\left[ \, \langle W(s),g\rangle _{L^2({\mathbb {R}} ^N)} \langle W(t),h \rangle _{L^2({\mathbb {R}} ^N)} \,\right] \\&\quad =\int _{{\mathbb {R}} ^N}\int _{{\mathbb {R}} ^N} \mathbb {E}\left[ \, W(s,x)W(t,y) \,\right] g(x)h(y)\,dxdy=(s \wedge t)\langle Q g,h\rangle _{L^2({\mathbb {R}} ^N)}, \end{aligned}$$

which is Eq. (2.3). Equation (2.18) is the reason why we stated in Sect. 2.1 that $W$ was a white noise in space and time.

### Examples

As mentioned we now present two important cases where the conditions (**C1’**) and (**C2’**) are satisfied. For convenience, in both cases we in fact show that $(\mathbf C1' )$ is satisfied for some $\rho _w\in L^1({\mathbb {R}} ^N)$ that is also bounded.

*Example 1*: $|w|$ defines a compact integral operator. Suppose that

- given $\varepsilon > 0$, there exists $\delta >0$ and $R>0$ such that for all $\theta \in {{\mathbb {R}} ^N}$ with $|\theta |<\delta$
  (i) for almost all $x\in {{\mathbb {R}} ^N}$,
    $$\begin{aligned} \int _{{{\mathbb {R}} ^N}\backslash B(0, R)} |w(x, y)| dy < \varepsilon , \quad \int _{{{\mathbb {R}} ^N}}|w(x, y+\theta )-w(x, y)|dy < \varepsilon , \end{aligned}$$
  (ii) for almost all $y\in {{\mathbb {R}} ^N}$,
    $$\begin{aligned} \int _{{{\mathbb {R}} ^N}\backslash B(0, R)} |w(x, y)| dx < \varepsilon , \quad \int _{{{\mathbb {R}} ^N}}|w(x+\theta , y)-w(x, y)|dx < \varepsilon , \end{aligned}$$
  where $B(0, R)$ denotes the ball of radius $R$ in ${{\mathbb {R}} ^N}$ centered at the origin;
- There exists a bounded subset $\varOmega \subset {{\mathbb {R}} ^N}$ of positive measure such that
  $$\begin{aligned} \inf _{y \in \varOmega }\int _\varOmega |w(x, y)|dx >0, \,\, \mathrm {or} \,\, \inf _{x \in \varOmega }\int _\varOmega |w(x, y)|dy >0; \end{aligned}$$
- $w$ satisfies (**C2’**) and moreover
  $$\begin{aligned} \forall y\in {{\mathbb {R}} ^N}\ (x\mapsto w(x, y))\in L^1({{\mathbb {R}} ^N}), \,\, \mathrm {and}\,\, \sup _{y\in {{\mathbb {R}} ^N}}\Vert w(\cdot , y)\Vert _{L^1({{\mathbb {R}} ^N})} < \infty . \end{aligned}$$

We claim that these assumptions are sufficient for (**C1’**) so that we can apply Theorem 2.2 in this case. Indeed, let $\mathbb {X}$ be the Banach space of functions in $L^1({{\mathbb {R}} ^N})\cap L^\infty ({{\mathbb {R}} ^N})$ equipped with the norm $\Vert \cdot \Vert _\mathbb {X} = \max \{\Vert \cdot \Vert _{L^1({{\mathbb {R}} ^N})}, \Vert \cdot \Vert _{L^\infty ({{\mathbb {R}} ^N})}\}$. Thanks to the last point above, we can well-define the map $J:\mathbb {X} \rightarrow \mathbb {X}$ by

$$\begin{aligned} Jh(y) = \int _{{\mathbb {R}} ^N} |w(x, y)|h(x)dx, \,\, h\in \mathbb {X}. \end{aligned}$$

Moreover, it follows from (Eveson (1995), Corollary 5.1) that the first condition we have here imposed on $w$ is in fact necessary and sufficient for both the operators $J:L^1({{\mathbb {R}} ^N})\rightarrow L^1({{\mathbb {R}} ^N})$ and $J:L^\infty ({{\mathbb {R}} ^N})\rightarrow L^\infty ({{\mathbb {R}} ^N})$ to be compact. We therefore clearly also have that the condition is necessary and sufficient for the operator $J:\mathbb {X}\rightarrow \mathbb {X}$ to be compact.

Note now that the space $\mathbb {K}$ of positive functions in $\mathbb {X}$ is a cone in $\mathbb {X}$ such that $J(\mathbb {K}) \subset \mathbb {K}$, and that the cone is *reproducing* (i.e. $\mathbb {X} = \{f - g: f, g \in \mathbb {K}\}$). If we can show that $r(J)$ is strictly positive, we can thus finally apply the Krein-Rutman Theorem [see for example (Du (2006), Theorem 1.1)] to see that $r(J)$ is an eigenvalue with corresponding non-zero eigenvector $\rho \in \mathbb {K}$.

To show that $r(J)>0$, suppose first of all that there exists a bounded $\varOmega \subset {{\mathbb {R}} ^N}$ of positive measure such that $\inf _{y \in \varOmega }\int _\varOmega |w(x, y)|dx >0$. Define $h=1$ on $\varOmega$, $0$ elsewhere, so that $\Vert h\Vert _{\mathbb {X}} = \max \{1, |\varOmega |\}$. Then, trivially,

$$\begin{aligned} \Vert Jh\Vert _{\mathbb {X}} \ge \sup _{y\in {{\mathbb {R}} ^N}} \int _\varOmega |w(x, y)|dx&\ge \inf _{y\in \varOmega }\int _{\varOmega } |w(x, y)|dx {=:} m >0, \end{aligned}$$

by assumption. Replacing $h$ by $\widetilde{h} = h/ \max \{1, |\varOmega |\}$ yields $\Vert \widetilde{h}\Vert _\mathbb {X}=1$ and

$$\begin{aligned} \Vert J\widetilde{h}\Vert _\mathbb {X} \ge m/ \max \{1, |\varOmega |\}. \end{aligned}$$

Thus $$\Vert J\Vert \ge m/ \max \{1, |\varOmega |\}$$. Similarly

$$\begin{aligned} \Vert J^2{h}\Vert _{\mathbb {X}}&\ge \sup _{y\in {{\mathbb {R}} ^N}}\int _{{\mathbb {R}} ^N} |w(x_1, y)| \left( \,\,\int _{\varOmega }|w(x_2, x_1)|dx_2\right) dx_1\\&\ge \int _{{\mathbb {R}} ^N} |w(x_1, y)| \left( \,\,\int _{\varOmega }|w(x_2, x_1)|dx_2\right) dx_1, \quad \forall y\in {{\mathbb {R}} ^N}\\&\ge \inf _{x_1\in \varOmega }\left( \,\,\int _{\varOmega }|w(x_2, x_1)|dx_2\right) \int _\varOmega |w(x_1, y)| dx_1, \quad \forall y\in {{\mathbb {R}} ^N}. \end{aligned}$$

Therefore

$$\begin{aligned} \Vert J^2{h}\Vert _{\mathbb {X}} \ge m^2, \end{aligned}$$

so that $\Vert J^2\Vert \ge m^2/\max \{1, |\varOmega |\}$. In fact we have $\Vert J^k\Vert \ge m^k/\max \{1, |\varOmega |\}$ for all $k\ge 1$, so that, by the spectral radius formula, $r(J)\ge m>0$. The case where $\inf _{x \in \varOmega }\int _\varOmega |w(x, y)|dy >0$ holds instead is proved similarly, by instead taking $h = 1/|\varOmega |$ on $\varOmega$ ($0$ elsewhere) and working with the $L^1({{\mathbb {R}} ^N})$ norm of $Jh$ in place of the $L^\infty ({{\mathbb {R}} ^N})$ norm.

We have thus found a non-negative, non-zero function $\rho = \rho _w \in L^1({{\mathbb {R}} ^N})\cap L^\infty ({{\mathbb {R}} ^N})$ such that

$$\begin{aligned} \int _{{\mathbb {R}} ^N} |w(x, y)|\rho _w(x)dx = r(J)\rho _w(y), \quad \forall y\in {{\mathbb {R}} ^N}, \end{aligned}$$

so that (**C1’**) is satisfied.

*Example 2*: Homogeneous case. Suppose that

- $w$ is homogeneous i.e $w(x,y) = w(x-y)$ for all $x, y\in {{\mathbb {R}} ^N}$;
- $w\in L^1({{\mathbb {R}} ^N})$ and is continuous;
- $$\int _{{\mathbb {R}} ^N}|x|^{2N}|w(x)|dx <\infty $$.

These conditions are satisfied for many typical choices of the neural field kernel in the literature [e.g. the “Mexican hat” kernel Bressloff et al. 2001; Faye et al. 2011; Owen et al. 2007; Veltz and Faugeras 2010 and (2.13) above]. However, it is clear that we are not in the case of the previous example, since for any $R>0$

$$\begin{aligned} \sup _{x\in {{\mathbb {R}} ^N}}\int _{{{\mathbb {R}} ^N}\backslash B(0, R)}|w(x-y)|dy = \Vert w\Vert _{L^1({{\mathbb {R}} ^N})}, \end{aligned}$$

which is not uniformly small. We thus again show that (**C1’**) is satisfied in this case so that [since (**C2’**) is trivially satisfied] Theorem 2.2 yields the existence of a unique $L^2({{\mathbb {R}} ^N}, \rho _w)$-valued solution to (2.5).

In order to do this, we use the Fourier transform. Let $v = |w|$, so that $v$ is continuous and in $L^1({{\mathbb {R}} ^N})$. Let $\mathfrak {F}v$ be the Fourier transform of $v$ i.e.

$$\begin{aligned} \mathfrak {F}v (\xi ) {:=} \int _{{\mathbb {R}} ^N} e^{-2\pi i x.\xi }v(x)dx, \quad \xi \in {{\mathbb {R}} ^N}. \end{aligned}$$

Therefore $\mathfrak {F}v$ is continuous and bounded by

$$\begin{aligned} \sup _{\xi \in {{\mathbb {R}} ^N}}|\mathfrak {F}v(\xi )| \le \Vert v\Vert _{L^1({{\mathbb {R}} ^N})} = \Vert w\Vert _{L^1({{\mathbb {R}} ^N})}. \end{aligned}$$

Now let $\Lambda _w = \Vert w\Vert _{L^1({{\mathbb {R}} ^N})} +1$, and $z(x) {:=} e^{-|x|^2/2}$, $x\in {{\mathbb {R}} ^N}$, so that $z$ is in the Schwartz space of smooth rapidly decreasing functions, which we denote by ${\mathcal {S}} ({{\mathbb {R}} ^N})$. Then define

2.19 $$\begin{aligned} \hat{\rho }(\xi ) {:=} \frac{\mathfrak {F}z(\xi )}{\Lambda _w - \mathfrak {F}v(\xi )}. \end{aligned}$$

We note that the denominator is continuous and strictly bounded away from $0$ (indeed by construction $\Lambda _w- \mathfrak {F}v(\xi ) \ge 1$ for all $\xi \in {{\mathbb {R}} ^N}$). Thus $\hat{\rho }$ is continuous, bounded and in $L^1({{\mathbb {R}} ^N})$ (since $\mathfrak {F}z\in {\mathcal {S}} ({{\mathbb {R}} ^N})$ by the standard stability result for the Fourier transform on ${\mathcal {S}} ({{\mathbb {R}} ^N})$).

We now claim that $\mathfrak {F}^{-1}\hat{\rho }(x) \in L^1({{\mathbb {R}} ^N})$, where the map $\mathfrak {F}^{-1}$ is defined by

$$\begin{aligned} \mathfrak {F}^{-1}g(x) {:=} \int _{{\mathbb {R}} ^N} e^{2\pi i x.\xi }g(\xi )d\xi , \quad g\in L^1({{\mathbb {R}} ^N}). \end{aligned}$$

Indeed, we note that for any $k\in \{1, \dots , N\}$,

$$\begin{aligned} \partial ^{2N}_{k}\mathfrak {F}v(\xi ) = (-2\pi i)^{2N} \int _{{\mathbb {R}} ^N} e^{-2\pi i x.\xi }x^{2N}_kv(x)dx, \end{aligned}$$

which is well-defined and bounded thanks to our assumption on the integrability of $x\mapsto |x|^{2N}|w(x)|$. Since $\mathfrak {F} z$ is rapidly decreasing, we can thus see that the function $\hat{\rho }(\xi )$ is $2N$ times differentiable with respect to every component and $\partial ^{2N}_{k}\hat{\rho }(\xi )$ is absolutely integrable for every $k\in \{1, \dots N\}$. Finally, since $\mathfrak {F}^{-1}(\partial ^{2N}_k\hat{\rho })(x) = (2\pi i)^{2N} x_k^{2N}\mathfrak {F}^{-1}\hat{\rho }(x)$ for each $k\in \{1, \dots , N\}$, we have that

$$\begin{aligned} |\mathfrak {F}^{-1}\hat{\rho }(x)| \le \frac{\sum _{k=1}^N |\mathfrak {F}^{-1}(\partial ^{2N}_k\hat{\rho })(x)|}{(2\pi )^{2N} \sum _{k=1}^Nx_k^{2N}} \le \frac{N^{N-1}\sum _{k=1}^N \Vert \partial ^{2N}_k\hat{\rho }\Vert _{L^1({\mathbb {R}} ^N)}}{(2\pi )^{2N} |x|^{2N}}, \end{aligned}$$

for all $x\in {\mathbb {R}} ^N$. Thus there exists a constant $K$ such that $|\mathfrak {F}^{-1}\hat{\rho }(x)| \le K/|x|^{2N}$. Moreover, since we also have the trivial bound

$$\begin{aligned} |\mathfrak {F}^{-1}\hat{\rho }(x)| \le \Vert \hat{\rho }\Vert _{L^1({{\mathbb {R}} ^N})}, \end{aligned}$$

for all $x\in {{\mathbb {R}} ^N}$, it follows that $|\mathfrak {F}^{-1}\hat{\rho }(x)| \le K/(1+|x|^{2N})$, by adjusting the constant $K$. Since this is integrable over ${\mathbb {R}} ^N$, the claim is proved.

Now, by the classical Fourier Inversion Theorem (which is applicable since $\hat{\rho }$ and $\mathfrak {F}^{-1}\hat{\rho }$ are both in $L^1({{\mathbb {R}} ^N})$), we thus have that

$$\begin{aligned} \mathfrak {F}\left( \mathfrak {F}^{-1}\hat{\rho }\right) (\xi ) = \hat{\rho }(\xi ), \end{aligned}$$

for all $$\xi \in {{\mathbb {R}} ^N}$$.

By setting $\rho (x) = \mathfrak {F}^{-1}\hat{\rho }(x)$, we see that

$$\begin{aligned} \Lambda _w\mathfrak {F}\rho (\xi ) - \mathfrak {F}\rho (\xi )\mathfrak {F}v(\xi ) {:=}\mathfrak {F}z(\xi ). \end{aligned}$$

We may finally again apply the inverse Fourier transform $\mathfrak {F}^{-1}$ to both sides, so that by the Inversion Theorem again (along with the standard convolution formula) it holds that

$$\begin{aligned} \Lambda _w\rho (y) - \int _{{{\mathbb {R}} ^N}}v(x-y)\rho (x)dx =e^{-\frac{|y|^2}{2}}, \quad y\in {{\mathbb {R}} ^N}. \end{aligned}$$

It then follows that

$$\begin{aligned} \int _{{{\mathbb {R}} ^N}}|w(x-y)|\rho (x)dx \le \Lambda _w\rho (y), \quad y\in {{\mathbb {R}} ^N}, \end{aligned}$$

as claimed.

Moreover, Eq. (2.19) shows that $\hat{\rho }(\xi )$ is in Schwartz space, hence so is $\rho$, implying that it is bounded. Note that Eq. (2.19) provides a way of explicitly computing one possible function $\rho _w$ appearing in condition (**C1’**) in the cases where the neural field kernel is homogeneous [for example given by (2.11) and (2.13)]. That particular function can be varied for example by changing the function $z$ and/or the constant $\Lambda _w$.

## Stochastic neural fields as Gaussian random fields

In this section we take an alternative approach, and try to give sense to a solution to the stochastic neural field Eq. (1.1) as a random field, using Walsh’s theory of integration.

This approach generally takes as its starting point a deterministic PDE, and then attempts include a term which is random in both space and time. With this in mind, consider first the well studied deterministic neural field equation

3.1 $$\begin{aligned} \partial _tY(t, x) = - Y(t, x) + \int _{{\mathbb {R}} ^N} w(x,y)G(Y(t, y))dy, \quad x\in {\mathbb {R}} ^N,\ t\ge 0. \end{aligned}$$

Under some conditions on the neural field kernel $w$ (boundedness, condition (**C2’**) above and $L^1$-Lipschitz continuity), this equation has a unique solution $(t, x)\mapsto Y(t, x)$ that is bounded and continuous in $x$ and continuously differentiable in $t$, whenever $x\mapsto Y(0, x)$ is bounded and continuous (Potthast 2010).

The idea then is to directly add a noise term to this equation, and try and give sense to all the necessary objects in order to be able to define what we mean by a solution. Indeed, consider the following stochastic version of (3.1),

3.2 $$\begin{aligned} \partial _tY(t, x) = - Y(t, x) + \int _{{\mathbb {R}} ^N} w(x, y)G(Y(t, y))dy + \sigma (Y(t, x))\dot{W}(t, x) \end{aligned}$$

where $\dot{W}$ is a “space-time white noise”. Informally we may think of the object $\dot{W}(t, x)$ as the random *distribution* which, when integrated against a test function $h\in L^2({\mathbb {R}} ^+\times {{\mathbb {R}} ^N})$

$$\begin{aligned} \dot{W}(h){:=} \int _0^\infty \int _{{\mathbb {R}} ^N} h(t, x)\dot{W}(t, x)dtdx, \quad h\in L^2({\mathbb {R}} ^+\times {{\mathbb {R}} ^N}), \end{aligned}$$

yields a zero-mean Gaussian random field $(\dot{W}(h))_{h\in L^2({\mathbb {R}} ^+\times {{\mathbb {R}} ^N})}$ with covariance

$$\begin{aligned} \mathbb {E}\left[ \, \dot{W}(g)\dot{W}(h) \,\right] = \int _0^\infty \int _{{\mathbb {R}} ^N} g(t, x) h(t, x) dxdt, \quad g, h\in L^2({\mathbb {R}} ^+\times {{\mathbb {R}} ^N}). \end{aligned}$$

The point is that with this interpretation of space-time white noise, since Eq. (3.2) specifies no regularity in the spatial direction (the map $x\mapsto Y(t,x)$ is simply assumed to be Lebesgue measurable so that the integral makes sense), it is clear that any solution will be *distribution*-valued in the spatial direction, which is rather unsatisfactory. Indeed, consider the extremely simple linear case when $G\equiv 0$ and $\sigma \equiv 1$, so that (3.2) reads

3.3 $$\begin{aligned} \partial _tY(t, x) = - Y(t, x) + \dot{W}(t, x). \end{aligned}$$

Formally, the solution to this equation is given by

$$\begin{aligned} Y(t, x) = e^{-t}Y(0, x) + \int _0^t e^{-(t-s)}\dot{W}(s, x) ds, \quad t\ge 0, x\in {{\mathbb {R}} ^N}, \end{aligned}$$

and since the integral is only over time it is clear (at least formally) that $x\mapsto Y(t, x)$ is a distribution for all $t\ge 0$. This differs significantly from the usual SPDE situation, when one would typically have an equation such as (3.3) where a second order differential operator in space is applied to the first term on the right-hand side (leading to the much studied *stochastic heat equation*). In such a case, the semigroup generated by the second order differential operator can be enough to smooth the space-time white noise in the spatial direction, leading to solutions that are continuous in both space and time [at least when the spatial dimension is $1$—see for example Pardoux (2007, Chapter 3) or Walsh (1986, Chapter 3)].

Of course one can develop a theory of distribution-valued processes [as is done in Walsh (1986, Chapter 4)] to interpret solutions of (3.2) in the obvious way: one says that the random field $(Y(t, x))_{t\ge 0, x\in {{\mathbb {R}} ^N}}$ is a (weak) solution to (3.2) if for all $\phi \in C_0^\infty ({{\mathbb {R}} ^N})$ it holds that

$$\begin{aligned} \int _{{{\mathbb {R}} ^N}}\phi (x) Y(t, x)dx&= e^{-t}\int _{{\mathbb {R}} ^N}\phi (x) Y(0, x)dx \\&\quad + \int _0^t\int _{{\mathbb {R}} ^N} e^{-(t-s)}\phi (x)\int _{{\mathbb {R}} ^N} w(x, y)G(Y(s, y))dydxds\\&\quad + \int _0^t\int _{{\mathbb {R}} ^N} e^{-(t-s)}\phi (x)\sigma (Y(s,x))\dot{W}(s, x)dxds, \end{aligned}$$

for all $t\ge 0$. Here all the integrals can be well-defined, which makes sense intuitively if we think of $\dot{W}(t, x)$ as a distribution. In fact it is more common to write $\int _0^t\int _{{\mathbb {R}} ^N} e^{-(t-s)}\phi (x)W(dsdx)$ for the stochastic integral term, once it has been rigorously defined.

However, we argue that it is not worth developing this theory here, since distribution valued solutions are of little interest physically. It is for this reason that we instead look for other types of random noise to add to the deterministic Eq. (3.1) which in particular will be correlated in space that will produce solutions that are real-valued random fields, and are at least Hölder continuous in both space and time. In the theory of SPDEs, when the spatial dimension is $2$ or more, the problem of an equation driven by space-time white noise having no real-valued solution is a well-known and much studied one [again see for example Pardoux (2007, Chapter 3) or Walsh (1986, Chapter 3) for a discussion of this]. To get around the problem, a common approach (Dalang and Frangos 1998; Ferrante and Sanz-Solé 2006; Sanz-Solé and Sarrà 2002) is to consider random noises that are smoother than white noise, namely a Gaussian noise that is white in time but has a smooth spatial covariance. Such random noise is known as either spatially colored or spatially homogeneous white-noise. One can then formulate conditions on the covariance function to ensure that real-valued Hölder continuous solutions to the specific SPDE exist.

It should also be mentioned, as remarked in Dalang and Frangos (1998), that in trying to model physical situations, there is some evidence that white-noise smoothed in the spatial direction is more natural, since spatial correlations are typically of a much larger order of magnitude than time correlations.

In the stochastic neural field case, since we have no second order differential operator, our solution will only ever be as smooth as the noise itself. We therefore look to add a noise term to (3.1) that is at least Hölder continuous in the spatial direction instead of pure white noise, and then proceed to look for solutions to the resulting equation in the sense of Walsh.

The section is structured as follows. First we briefly introduce Walsh’s theory of stochastic integration, for which the classical reference is Walsh (1986). This theory will be needed to well-define the stochastic integral in our definition of a solution to the neural field equation. We then introduce the spatially smoothed space-time white noise that we will consider, before finally applying the theory to analyze solutions of the neural field equation driven by this spatially smoothed noise under certain conditions.

### Walsh’s stochastic integral

We will not go into the details of the construction of Walsh’s stochastic integral, since a very nice description is given by D. Khoshnevisan in Dalang et al. (2009) [see also Walsh (1986)]. Instead we present the bare essentials needed in the following sections.

The elementary object of study is the centered Gaussian random field^6^

$$\begin{aligned} \dot{W} {:=} (\dot{W}(A))_{A\in \mathcal {B}({\mathbb {R}} ^+\times {{\mathbb {R}} ^N})} \end{aligned}$$

indexed by $A\in \mathcal {B}({\mathbb {R}} ^+\times {{\mathbb {R}} ^N})$ (where ${\mathbb {R}} ^{+} {:=} [0, \infty )$) with covariance function

3.4 $$\begin{aligned} \mathbb {E}\left[ \, \dot{W}(A)\dot{W}(B) \,\right] =|A\cap B|, \,\, A, B, \in \mathcal {B}({\mathbb {R}} ^+\times {{\mathbb {R}} ^N}), \end{aligned}$$

where $|A\cap B|$ denotes the Lebesgue measure of $A\cap B$. We say that $\dot{W}$ is a *white noise* on ${\mathbb {R}} ^+\times {{\mathbb {R}} ^N}$. We then define the *white noise process* $W{:=}(W_t(A))_{t\ge 0, A\in \mathcal {B}({{\mathbb {R}} ^N})}$ by

3.5 $$\begin{aligned} W_t(A){:=} \dot{W}([0, t]\times A), \quad t\ge 0. \end{aligned}$$

Now define the norm

3.6 $$\begin{aligned} \Vert f\Vert ^2_W&{:=} {\mathbb {E}} \left[ \int _0^T\int _{{{\mathbb {R}} ^N}} |f(t, x)|^2dtdx\right] , \end{aligned}$$

for any (random) function $f$ that is knowable^7^ at time $t$ given $(W_s(A))_{s\le t, A\in {\mathcal {B}}({{\mathbb {R}} ^N})}$. Then let $\mathfrak {P}_W$ be the set of all such functions $f$ for which $\Vert f\Vert _{W} <\infty$. The point is that this space forms the set of integrands that can be integrated against the white noise process according to Walsh’s theory.

Indeed, we have then following theorem ((Walsh 1986, Theorem 2.5)).

#### **Theorem 3.1**

For all $f\in \mathfrak {P}_W$, $t\in [0,T]$ and $A\in {\mathcal {B}} ({{\mathbb {R}} ^N})$,

$$\begin{aligned} \int _0^t\int _Af(s, x)W(dsdx) \end{aligned}$$

can be well-defined in $L^2(\varOmega , {\mathcal {F}} , \mathbb {P})$. Moreover for all $t\in (0, T]$ and $A, B\in {\mathcal {B}} ({{\mathbb {R}} ^N})$, ${\mathbb {E}} [\int _0^t\int _Af(s, x)W(dsdx)] =0$ and

$$\begin{aligned} {\mathbb {E}} \left[ \int _0^t\int _Af(s, x)W(dsdx) \int _0^t\int _Bf(s, x)W(dsdx)\right] = {\mathbb {E}} \left[ \int _0^t\int _{A\cap B}f^2(s,x)dxdt\right] \!. \end{aligned}$$

The following inequality will also be fundamental:

#### **Theorem 3.2**

(Burkhölder’s inequality) For all $p\ge 2$ there exists a constant $c_p$ (with $c_2 =1$) such that for all $f\in \mathfrak {P}_W$, $t\in (0, T]$ and $A\in {\mathcal {B}} ({{\mathbb {R}} ^N})$,

$$\begin{aligned} {\mathbb {E}} \left[ \left| \int _0^t\int _Af(s, x)W(dsdx)\right| ^p\right] \le c_p{\mathbb {E}} \left[ \left( \int _0^T\int _{{{\mathbb {R}} ^N}}|f(t, x)|^2dtdx\right) ^\frac{p}{2}\right] \!. \end{aligned}$$

### Spatially smoothed space-time white noise

Let $W = (W_t(A))_{t\ge 0, A\in {\mathcal {B}} ({{\mathbb {R}} ^N})}$ be a white-noise process as defined in the previous section. For $\varphi \in L^2({{\mathbb {R}} ^N})$, we can well-define the (Gaussian) random field $(W^\varphi (t, x))_{t\ge 0, x\in {{\mathbb {R}} ^N}}$ for any $T>0$ by

3.7 $$\begin{aligned} W^\varphi (t, x) {:=} \int _0^t \int _{{\mathbb {R}} ^N} \varphi (x-y)W(dsdy). \end{aligned}$$

To see this one just needs to check that $\varphi (x - \cdot )\in \mathfrak {P}_W$ for every $x$, where $\mathfrak {P}_W$ is as above. The function $\varphi (x - \cdot )$ is clearly completely determined by $W$ for each $x$ (since it is non-random) and for every $T>0$

$$\begin{aligned} \Vert \varphi (x - \cdot )\Vert _W^2&= {\mathbb {E}} \left[ \int _0^T\int _{{{\mathbb {R}} ^N}}|\varphi (x-z)|^2dtdz \right] \\&= T\Vert \varphi \Vert _{L^2({{\mathbb {R}} ^N})}^2 < \infty , \end{aligned}$$

so that the integral in (3.7) is indeed well-defined in the sense of the above construction. Moreover, by Theorem 3.1 the random field $(W^\varphi (t, x))_{t\ge 0, x\in {\mathbb {R}} ^N}$ has spatial covariance

$$\begin{aligned} {\mathbb {E}} [W^\varphi (t, x)W^\varphi (t, y)]&= {\mathbb {E}} \left[ \int _0^t \int _{{\mathbb {R}} ^N} \varphi (x-z)\,W(dsdz) \int _0^t \int _{{\mathbb {R}} ^N} \varphi (y-z)\,W(dsdz)\right] \\&= \int _0^t \int _{{\mathbb {R}} ^N} \varphi (x-z)\varphi (y-z)dzds = t\varphi \star \widetilde{\varphi } (x-y), \end{aligned}$$

where $\star$ denotes the convolution operator as usual, and $\widetilde{\varphi }(x)=\varphi (-x)$. Thus the random field $(W^\varphi (t, x))_{t\ge 0, x\in {\mathbb {R}} ^N}$ is spatially correlated.

The regularity in time of this process is the same as that of a Brownian path:

#### **Lemma 3.3**

For any $x\in {{\mathbb {R}} ^N}$, the path $t\mapsto W^\varphi (t, x)$ has an $\eta$-Hölder continuous modification for any $\eta \in (0, 1/2)$.

#### *Proof*

For $x\in {{\mathbb {R}} ^N}$, $s,t\ge 0$ with $s\le t$ and any $p\ge 2$ we have by Burkhölder’s inequality (Theorem 3.2 above) that

$$\begin{aligned} \mathbb {E}\left[ \, \left| W^\varphi (t, x) - W^\varphi (s, x)\right| ^p \,\right] \le c_p\Vert \varphi \Vert _{L^2({{\mathbb {R}} ^N})}^2(t-s)^\frac{p}{2}. \end{aligned}$$

The result follows from the standard Kolmogorov continuity theorem [see for example Theorem 4.3 of Dalang et al. (2009, Chapter 1)].$\square$

More importantly, if we impose some (very weak) regularity on $\varphi$ then $W^\varphi$ inherits some spatial regularity:

#### **Lemma 3.4**

Suppose that there exists a constant $C_\varphi$ such that

3.8 $$\begin{aligned} \Vert \varphi - {\varvec{\tau }}_{z}(\varphi )\Vert _{L^2({{\mathbb {R}} ^N})} \le C_\varphi |z|^\alpha , \quad \forall z\in {{\mathbb {R}} ^N}, \end{aligned}$$

for some $\alpha \in (0, 1]$, where ${\varvec{\tau }}_{z}$ indicates the shift by $z$ operator (so that ${\varvec{\tau }}_z(\varphi )(y){:=}\varphi (y+z)$ for all $y, z\in {{\mathbb {R}} ^N}$). Then for all $t\ge 0$, the map $x\mapsto W^\varphi (t, x)$ has an $\eta$-Hölder continuous modification, for any $\eta \in (0, \alpha )$.

#### *Proof*

For $x, \widetilde{x} \in {{\mathbb {R}} ^N}$, $t\ge 0$, and any $p\ge 2$ we have (again by Burkhölder’s inequality) that

$$\begin{aligned} \mathbb {E}\left[ \, \left| W^\varphi (t, x) - W^\varphi (t, \widetilde{x})\right| ^p \,\right]&\le t^\frac{p}{2}c_p\left( \,\,\int _{{\mathbb {R}} ^N} |\varphi (x-y) - \varphi (\widetilde{x}-y)|^2dy\right) ^\frac{p}{2}\\&= t^\frac{p}{2}c_p\left( \,\,\int _{{\mathbb {R}} ^N} |\varphi (y) - \varphi (y+\widetilde{x} - x)|^2dy\right) ^\frac{p}{2}\\&\le t^\frac{p}{2}c_pC_\varphi ^p|x -\widetilde{x}|^{p\alpha }. \end{aligned}$$

The result follows by Kolmogorov’s continuity theorem.$\square$

#### *Remark 3.5*

The condition (3.8) with $\alpha =1$ is true if and only if the function $\varphi$ is in the Sobolev space $W^{1, 2}({{\mathbb {R}} ^N})$ ((Brezis 2010, Proposition 9.3)).

When $\alpha <1$ the set of functions $\varphi \in L^2({{\mathbb {R}} ^N})$ which satisfy (3.8) defines a Banach space denoted by $N^{\alpha , 2}({{\mathbb {R}} ^N})$ which is known as the Nikolskii space. This space is closely related to the more familiar fractional Sobolev space $W^{\alpha , 2}({{\mathbb {R}} ^N})$ though they are not identical. We refer to Simon (1990) for a detailed study of such spaces and their relationships. An example of when (3.8) holds with $\alpha =1/2$ is found by taking $\varphi$ to be an indicator function. It is in this way we see that (3.8) is a rather weak condition.

### The stochastic neural field equation driven by spatially smoothed space-time white noise

We now have everything in place to define and study the solution to the stochastic neural field equation driven by a spatially smoothed space-time white noise. Indeed, consider the equation

3.9 $$\begin{aligned} \partial _tY(t, x) = - Y(t, x) + \int _{{\mathbb {R}} ^N} w(x,y)G(Y(t, y))dy + \sigma (Y(t, x))\frac{\partial }{\partial t}W^\varphi (t, x),\nonumber \\ \end{aligned}$$

with initial condition $Y(0, x) = Y_0(x)$ for $x\in {{\mathbb {R}} ^N}$ and $t\ge 0$, where $(W^\varphi (t, x))_{t\ge 0, x\in {{\mathbb {R}} ^N}}$ is the spatially smoothed space-time white noise defined by (3.7) for some $\varphi \in L^2({{\mathbb {R}} ^N})$. As above, we will impose Lipschitz assumptions on $\sigma$ and $G$, by supposing that

- $\sigma :{\mathbb {R}} \rightarrow {\mathbb {R}}$ is globally Lipschitz [exactly as in (2.15)] i.e. there exists a constant $C_\sigma$ such that
  $$\begin{aligned} |\sigma (a) - \sigma (b)| \le C_\sigma |a-b|, \ \mathrm {and} \ \ |\sigma (a)| \le C_\sigma (1 + |a|), \quad \forall a, b\in {\mathbb {R}} ; \end{aligned}$$
- $G:{\mathbb {R}} \rightarrow {\mathbb {R}}$ is bounded and globally Lipschitz (exactly as above) i.e. such that there exists a constant $C_G$ with $\sup _{a\in {\mathbb {R}} }|G(a)| \le C_G$ and
  $$\begin{aligned} |G(a) - G(b)| \le C_G|a- b|, \quad \forall a, b \in {\mathbb {R}} . \end{aligned}$$

Although the above equation is not well-defined ($\frac{\partial }{\partial t}W^\varphi (t, x)$ does not exist), we will interpret a solution to (3.9) in the following way.

#### **Definition 3.6**

By a solution to (3.9) we will mean a real-valued random field $(Y(t, x))_{t\ge 0, x\in {{\mathbb {R}} ^N}}$ such that

3.10 $$\begin{aligned} Y(t, x)&= e^{-t}Y_0(x) + \int _0^te^{-(t-s)}\int _{{\mathbb {R}} ^N} w(x, y)G(Y(s, y))dyds \nonumber \\&+ \int _0^t\int _{{{\mathbb {R}} ^N}}e^{-(t-s)}\sigma (Y(s, x))\varphi (x-y)W(dsdy), \end{aligned}$$

almost surely for all $t\ge 0$ and $x\in {{\mathbb {R}} ^N}$, where the stochastic integral term is understood in the sense described in Sect. 3.1.

Once again we are interested in the conditions on the neural field kernel $w$ that allow us to prove the existence of solutions in this new sense. Recall that in Sect. 2 we either required conditions (**C1**) and (**C2**) or (**C1’**) and (**C2’**) to be satisfied. The difficulty was to keep everything well-behaved in the Hilbert space $L^2({{\mathbb {R}} ^N})$ (or $L^2({{\mathbb {R}} ^N}, \rho )$). However, when looking for solutions in the sense of random fields $(Y(t, x))_{t\ge 0, x\in {{\mathbb {R}} ^N}}$ such that (3.10) is satisfied, such restrictions are no longer needed, principally because we no longer have to concern ourselves with the behavior in space at infinity. Indeed, in this section we simply work with the condition (**C2’**) i.e. that

$$\begin{aligned} \forall x\in {{\mathbb {R}} ^N}\ (y\mapsto w(x, y))\in L^1({{\mathbb {R}} ^N}), \,\, \mathrm {and}\quad \sup _{x\in {{\mathbb {R}} ^N}}\Vert w(x,\cdot )\Vert _{L^1({{\mathbb {R}} ^N})} \le C_w, \end{aligned}$$

for some constant $C_w$. Using the standard technique of a Picard iteration scheme [closely following Walsh (1986, Theorem 3.2)] and the simple properties of the Walsh stochastic integral stated in Sect. 3.1, we can prove the following:

#### **Theorem 3.7**

Suppose that the map $x\mapsto Y_0(x)$ is Borel measurable almost surely, and that

$$\begin{aligned} \sup _{x\in {{\mathbb {R}} ^N}}\mathbb {E}\left[ \, |Y_0(x)|^2 \,\right] < \infty . \end{aligned}$$

Suppose moreover that the neural field kernel $w$ satisfies condition (**C2’**). Then there exists an almost surely unique predictable random field $(Y(t, x))_{t\ge 0, x\in {{\mathbb {R}} ^N}}$ which is a solution to (3.9) in the sense of Definition 3.6 such that

3.11 $$\begin{aligned} \sup _{t\in [0, T], x\in {{\mathbb {R}} ^N}}\mathbb {E}\left[ \, |Y(t, x)|^2 \,\right] < \infty , \end{aligned}$$

for any $$T>0$$.

#### *Proof*

The proof proceeds in a classical way, but where we are careful to interpret all stochastic integrals as described in Sect. 3.1, and so we provide the details.

*Uniqueness*: Suppose that $(Y(t, x))_{t\ge 0, x\in {{\mathbb {R}} ^N}}$ and $(Z(t, x))_{t\ge 0, x\in {{\mathbb {R}} ^N}}$ are both solutions to (3.9) in the sense of Definition 3.6. Let $D(t, x) = Y(t, x) - Z(t, x)$ for $x\in {{\mathbb {R}} ^N}$ and $t\ge 0$. Then we have

$$\begin{aligned} D(t, x)&= \int _0^te^{-(t-s)}\int _{{\mathbb {R}} ^N} w(x, y)[G(Y(s, y)) - G(Z(s, y))]dyds \\&\quad + \int _0^t\int _{{{\mathbb {R}} ^N}}e^{-(t-s)}[\sigma (Y(s, x)) - \sigma (Z(s, x))]\varphi (x-y)W(dsdy). \end{aligned}$$

Therefore

$$\begin{aligned}&\mathbb {E}\left[ \, |D(t, x)|^2 \,\right] \\&\quad \le 2\mathbb {E}\left[ \, \left( \int _0^te^{-(t-s)}\int _{{\mathbb {R}} ^N} |w(x, y)||G(Y(s, y)) - G(Z(s, y))|dyds\right) ^2 \,\right] \\&\quad + 2\mathbb {E}\left[ \, \left( \int _0^t\int _{{{\mathbb {R}} ^N}}e^{-(t-s)}[\sigma (Y(s, x)) - \sigma (Z(s, x))]\varphi (x-y)W(dsdy)\right) ^2 \,\right] \\&\le 2t\int _0^te^{-2(t-s)}\mathbb {E}\left[ \, \left( \int _{{\mathbb {R}} ^N} |w(x, y)||G(Y(s, y)) - G(Z(s, y))|dy\right) ^2 \,\right] ds \\&\quad + 2\int _0^t\int _{{{\mathbb {R}} ^N}}e^{-2(t-s)}\mathbb {E}\left[ \, |\sigma (Y(s, x)) - \sigma (Z(s, x))|^2 \,\right] |\varphi (x-y)|^2dsdy, \end{aligned}$$

where we have used Cauchy–Schwarz and Burkhölder’s inequality (Theorem 3.2) with $p=2$. Thus, using the Lipschitz property of $\sigma$ and $G$,

$$\begin{aligned} \mathbb {E}\left[ \, |D(t, x)|^2 \,\right]&\le \!2tC_G^2\int _0^te^{-2(t-s)}\mathbb {E}\left[ \, \left( \int _{{\mathbb {R}} ^N} |w(x, y)||D(s, y)|dy\right) ^2 \,\right] ds \\&\quad + 2C_\sigma ^2\Vert \varphi \Vert ^2_{L^2({{\mathbb {R}} ^N})}\int _0^te^{-2(t-s)}\mathbb {E}\left[ \, |D(s, x)|^2 \,\right] ds. \end{aligned}$$

By the Cauchy–Schwarz inequality once again

$$\begin{aligned} \mathbb {E}\left[ \, |D(t, x)|^2 \,\right]&\le \! 2tC_G^2\Vert w(x, \cdot )\Vert _{L^1({{\mathbb {R}} ^N})}\! \int _0^te^{-2(t-s)}\!\int _{{\mathbb {R}} ^N} |w(x, y)|\,\mathbb {E}\left[ \, |D(s, y)|^2 \,\right] dyds \\&\quad + 2C_\sigma ^2\Vert \varphi \Vert ^2_{L^2({{\mathbb {R}} ^N})}\int _0^te^{-2(t-s)}\mathbb {E}\left[ \, |D(s, x)|^2 \,\right] ds. \end{aligned}$$

Let $H(s) {:=} \sup _{x\in {{\mathbb {R}} ^N}}{\mathbb {E}} [| D(s, x)|^2]$, which is finite since we are assuming $Y$ and $Z$ satisfy (3.11). Writing $K=2\max \{C^2_\sigma , C^2_G\}$, we have

$$\begin{aligned} \mathbb {E}\left[ \, |D(t, x)|^2 \,\right]&\le K\left[ tC_w^2 + \Vert \varphi \Vert _{L^2({{\mathbb {R}} ^N})}^2\right] \int _0^t e^{-2(t-s)}H(s)ds \\ \Rightarrow H(t)&\le K\left[ tC_w^2 + \Vert \varphi \Vert _{L^2({{\mathbb {R}} ^N})}^2\right] \int _0^t H(s)ds. \end{aligned}$$

An application of Gronwall’s lemma then yields $\sup _{s\le t}H(s) = 0$ for all $t\ge 0$. Hence $Y(t, x) = Z(t, x)$ almost surely for all $t\ge 0, x\in {{\mathbb {R}} ^N}$.

*Existence:* Let $Y_0(t, x) = Y_0(x)$. Then define iteratively for $n\in \mathbb {N}_0$, $t\ge 0$, $x\in {{\mathbb {R}} ^N}$,

3.12 $$\begin{aligned} Y_{n+1}(t, x)&{:=}\, e^{-t} Y_0(x) + \int _0^te^{-(t-s)}\int _{{\mathbb {R}} ^N} w(x, y)G(Y_n(s, y))dyds \nonumber \\&\quad + \int _0^t\int _{{{\mathbb {R}} ^N}}e^{-(t-s)}\sigma (Y_n(s, x))\varphi (x-y)W(dsdy). \end{aligned}$$

We first check that the stochastic integral is well-defined, under the assumption that

3.13 $$\begin{aligned} \sup _{t\in [0, T], x\in {{\mathbb {R}} ^N}}{\mathbb {E}} (|Y_n(t, x)|^2) <\infty , \end{aligned}$$

for any $T>0$, which we know is true for $n=0$ by assumption, and we show by induction is also true for each integer $n\ge 1$ below. To this end for any $T>0$

$$\begin{aligned}&{\mathbb {E}} \left[ \int _0^T\int _{{{\mathbb {R}} ^N}}e^{-2(t-s)}\sigma ^2(Y_n(s, x))\varphi ^2(x-y)dsdy\right] \\&\qquad \le 2C_\sigma ^2\Vert \varphi \Vert ^2_{L^2({{\mathbb {R}} ^N})}\int _0^T(1 + {\mathbb {E}} \left[ |Y_n(s, x)|^2\right] )ds\\&\qquad \le 2C_\sigma ^2\Vert \varphi \Vert ^2_{L^2({{\mathbb {R}} ^N})}T\left[ 1 + \sup _{t\in [0, T], x\in {{\mathbb {R}} ^N}}\mathbb {E}\left[ \, |Y_n(t, x)|^2 \,\right] \right] <\infty . \end{aligned}$$

This shows that the integrand in the stochastic integral is in the space $\mathfrak {P}_W$ (for all $T>0$), which in turn implies that the stochastic integral in the sense of Walsh is indeed well-defined (by Theorem 3.1).

Now define $D_n(t, x) {:=} Y_{n+1}(t, x) - Y_{n}(t, x)$ for $n\in \mathbb {N}_0$, $t\ge 0$ and $x\in {{\mathbb {R}} ^N}$. Then exactly as in the uniqueness calculation we have

$$\begin{aligned} \mathbb {E}\left[ \, |D_{n}(t, x)|^2 \,\right]&\le 2tC^2_GC_w\int _0^te^{-2(t-s)}\int _{{\mathbb {R}} ^N}|w(x,y)|\mathbb {E}\left[ \, \left| D_{n-1}(s, y)\right| ^2 \,\right] dyds\\&\quad + 2C^2_\sigma \Vert \varphi \Vert _{L^2({{\mathbb {R}} ^N})}^2 \int _0^t\mathbb {E}\left[ \, \left| D_{n-1}(s,x)\right| ^2 \,\right] e^{-2(t-s)}ds . \end{aligned}$$

This implies that by setting $H_n(s) = \sup _{x\in {{\mathbb {R}} ^N}} \mathbb {E}\left[ \, \left| D_{n}(s, x)\right| ^2 \,\right]$,

3.14 $$\begin{aligned} H_n(t)&\le K^n\left[ tC_w^2 + \Vert \varphi \Vert _{L^2({{\mathbb {R}} ^N})}^2\right] ^n \int _0^t\int _0^{t_1}\dots \int _0^{t_{n-1}}H_{0}(t_n)dt_n\dots dt_1, \end{aligned}$$

for all $n\in \mathbb {N}_0$ and $t\ge 0$. Now, similarly, we can find a constant $C_t$ such that

$$\begin{aligned}&\mathbb {E}\left[ \, \left| D_0(s, x)\right| ^2 \,\right] \le C_t\left( 1 + \sup _{x\in {{\mathbb {R}} ^N}} \mathbb {E}\left[ \, |Y_0(x)|^2 \,\right] \right) , \end{aligned}$$

for any $x\in {{\mathbb {R}} ^N}$ and $s\in [0, t]$, so that for $s\in [0, t]$,

$$\begin{aligned} H_0(s) = \sup _{x\in {{\mathbb {R}} ^N}} \mathbb {E}\left[ \, \left| D_{0}(s, x) \right| ^2 \,\right] \le C_t \left( 1 + \sup _{x\in {{\mathbb {R}} ^N}} \mathbb {E}\left[ \, |Y_0(x)|^2 \,\right] \right) , \end{aligned}$$

Using this in (3.14) we see that,

$$\begin{aligned} H_n(t) \le {C}_tK^{n}\left[ tC_w^2 + \Vert \varphi \Vert _{L^2({{\mathbb {R}} ^N})}^2\right] ^{n} \left( 1 + \sup _{x\in {{\mathbb {R}} ^N}} \mathbb {E}\left[ \, |Y_0(x)|^2 \,\right] \right) \frac{t^n}{n!}, \end{aligned}$$

for all $t\ge 0$. This is sufficient to see that (3.13) holds uniformly in $n$.

By completeness, for each $t\ge 0$ and $x\in {{\mathbb {R}} ^N}$ there exists $Y(t, x)\in L^2(\varOmega , {\mathcal {F}} , \mathbb {P})$ such that $Y(t, x)$ is the limit in $L^2(\varOmega , {\mathcal {F}} , \mathbb {P})$ of the sequence of square-integrable random variables $(Y_n(t, x))_{n\ge 1}$. Moreover, the convergence is uniform on $[0, T]\times {{\mathbb {R}} ^N}$, i.e.

$$\begin{aligned} \sup _{t\in [0, T], x\in {{\mathbb {R}} ^N}} {\mathbb {E}} \left| Y_n(t, x) - Y(t,x)\right| ^2\rightarrow 0. \end{aligned}$$

From this we can see that (3.11) is satisfied for the random field $(Y(t, x))_{t\ge 0, x\in {{\mathbb {R}} ^N}}$. It remains to show that $(Y(t, x))_{t\ge 0, x\in {{\mathbb {R}} ^N}}$ satisfies (3.10) almost surely. By the above uniform convergence, we have that

$$\begin{aligned} \mathbb {E}\left[ \, \left| \int _0^t\int _{{{\mathbb {R}} ^N}}e^{-(t-s)} \left[ \sigma (Y_n(s,x)) - \sigma (Y(s,x)) \right] \varphi (x-y)W(dsdy)\right| ^2 \,\right] \rightarrow 0, \end{aligned}$$

and

$$\begin{aligned} \mathbb {E}\left[ \, \left| \int _0^te^{-(t-s)} \int _{{{\mathbb {R}} ^N}}w(x, y)\left[ G(Y_n(s,y)) - G(Y(s,y)) \right] dsdy\right| ^2 \,\right] \rightarrow 0, \end{aligned}$$

uniformly for all $t\ge 0$ and $x\in {{\mathbb {R}} ^N}$. Thus taking the limit as $n\rightarrow \infty$ in (3.12) [in the $L^2(\varOmega , {\mathcal {F}} , \mathbb {P})$ sense] proves that $(Y(t, x))_{t\ge 0, x\in {{\mathbb {R}} ^N}}$ does indeed satisfy (3.10) almost surely.$\square$

In a very similar way, one can also prove that the solution remains $L^p$-bounded whenever the initial condition is $L^p$-bounded for any $p>2$. Moreover, this also allows us to conclude that the solution has time continuous paths for all $x\in {{\mathbb {R}} ^N}$.

#### **Theorem 3.8**

Suppose that we are in the situation of Theorem 3.7, but in addition we have that $\sup _{x\in {{\mathbb {R}} ^N}}\mathbb {E}\left[ \, |Y_0(x)|^p \,\right] <\infty$ for some $p>2$. Then the solution $(Y(t,x))_{t\ge 0, x\in {{\mathbb {R}} ^N}}$ to (3.9) in the sense of Definition 3.6 is $L^p$-bounded on $[0,T]\times {{\mathbb {R}} ^N}$ for any $T$ i.e.

$$\begin{aligned} \sup _{t\in [0, T],x\in {{\mathbb {R}} ^N}}\mathbb {E}\left[ \, \left| Y(t, x)\right| ^p \,\right] <\infty , \end{aligned}$$

and the map $t\mapsto Y(t, x)$ has a continuous version for all $x\in {{\mathbb {R}} ^N}$.

If the initial condition has finite $p$-moments for all $p>2$, then $t\mapsto Y(t, x)$ has an $\eta$-Hölder continuous version, for any $\eta \in (0, 1/2)$ and any $x\in {{\mathbb {R}} ^N}$.

#### *Proof*

The proof of the first part of this result uses similar techniques as in the proof of Theorem 3.7 in order to bound $\mathbb {E}\left[ \, |Y(t, x)|^p \,\right]$ uniformly in $t\in [0, T]$ and $x\in {{\mathbb {R}} ^N}$. In particular, we use the form of $Y(t, x)$ given by (3.10), Burkhölder’s inequality (see Theorem 3.2), Hölder’s inequality and Gronwall’s lemma, as well as the conditions imposed on $w$, $\sigma$, $G$ and $\varphi$.

For the time continuity, we again use similar techniques to achieve the bound

$$\begin{aligned} \mathbb {E}\left[ \, \left| Y(t, x) - Y(s, x)\right| ^p \,\right] \le C_T^{( p)}\left( 1 + \sup _{r\in [0, T],y\in {{\mathbb {R}} ^N}}\mathbb {E}\left[ \, |Y(r, y)|^p \,\right] \right) (t-s)^\frac{p}{2}, \end{aligned}$$

for all $s,t\in [0, T]$ with $s\le t$ and $x\in {{\mathbb {R}} ^N}$, for some constant $C_T^{(p)}$. The results then follow from Kolmogorov’s continuity theorem once again.$\square$

#### Spatial regularity of solution

As mentioned in the introduction to this section, the spatial regularity of the solution $(Y(t, x))_{t\ge 0, x\in {{\mathbb {R}} ^N}}$ to (3.9) is of interest. In particular we would like to find conditions under which it is at least continuous in space. As we saw in Lemma 3.4, under the weak condition on $\varphi$ given by (3.8), we have that the spatially smoothed space-time white noise is continuous in space. We here show that under this assumption together with a Hölder continuity type condition on the neural field kernel $w$, the solution $(Y(t, x))_{t\ge 0, x\in {{\mathbb {R}} ^N}}$ inherits the spatial regularity of the the driving noise.

It is worth mentioning that the neural field equation fits into the class of degenerate diffusion SPDEs (indeed there is no diffusion term), and that regularity theory for such equations is an area that is currently very active [see for example Hofmanová (2013) and references therein]. However, in our case we are not concerned with any kind of sharp regularity results [in contrast to those found in Dalang and Sanz-Solé (2009) for the stochastic wave equation], and simply want to assert that for most typical choices of neural field kernels $w$ made by practitioners, the random field solution to the neural field equation is at least regular in space. The results of the section are simple applications of standard techniques to prove continuity in space of random field solutions to SPDEs, as is done for example in Walsh (1986, Corollary 3.4).

The condition we introduce on $w$ is the following:

$$\begin{aligned} \exists K_w \ge 0 \ \ \mathrm {s.t.}\ \ \Vert w(x, \cdot ) - w(\widetilde{x}, \cdot )\Vert _{L^1({{\mathbb {R}} ^N})} \le L_w |x-\widetilde{x}|^\alpha , \quad \forall x, \widetilde{x} \in {{\mathbb {R}} ^N},\quad (\mathbf C3' ) \end{aligned}$$

for some $$\alpha \in (0, 1]$$.

##### *Remark 3.9*

This condition is certainly satisfied for all typical choices of neural field kernel $w$. In particular, any smooth rapidly decaying function will satisfy $(\mathbf{C3'})$.

##### **Theorem 3.10**

(Regularity) Suppose that we are in the situation of Theorem 3.7 and

$$\begin{aligned} \sup _{x\in {{\mathbb {R}} ^N}}\mathbb {E}\left[ \, |Y_0(x)|^p \,\right] < \infty \end{aligned}$$

for all $p\ge 2$. Suppose moreover that there exists $\alpha \in (0, 1]$ such that

- $$w$$ satisfies (**C3’**);
- $$\varphi $$ satisfies (3.8) i.e.
  $$\begin{aligned} \Vert \varphi - {\varvec{\tau }}_z(\varphi )\Vert _{L^2({{\mathbb {R}} ^N})} \le C_\varphi |z|^\alpha , \quad \forall z\in {{\mathbb {R}} ^N}, \end{aligned}$$
  where ${\varvec{\tau }}_z$ indicates the shift by $z\in {{\mathbb {R}} ^N}$ operator;
- $x\mapsto Y_0(x)$ is $\alpha$-Hölder continuous.

Then $(Y(t, x))_{t\ge 0, x\in {{\mathbb {R}} ^N}}$ has a modification such that $(t, x)\mapsto Y(t,x)$ is $(\eta _1, \eta _2)$-Hölder continuous, for any $\eta _1 \in (0,1/2)$ and $\eta _2 \in (0,\alpha )$.

##### *Proof*

Let $(Y(t, x))_{t\ge 0, x\in {{\mathbb {R}} ^N}}$ be the mild solution to (3.9), which exists and is unique by Theorem 3.7. The stated regularity in time is given in Theorem 3.8. It thus remains to prove the regularity in space.

Let $t\ge 0$, $x\in {{\mathbb {R}} ^N}$. Then by (3.10)

3.15 $$\begin{aligned} Y(t, x)&= e^{-t}Y_0(x) + I_1(t, x) + I_2(t, x), \end{aligned}$$

for all $t\ge 0$ and $x\in {{\mathbb {R}} ^N}$, where $I_1(t, x) = \int _0^te^{-(t-s)}\int _{{\mathbb {R}} ^N} w(x, y)G(Y(s, y))dyds$ and $I_2(t, x) = \int _0^t\int _{{{\mathbb {R}} ^N}}e^{-(t-s)}\sigma (Y(s, x))\varphi (x-y)W(dsdy)$.

Now let $p\ge 2$. The aim is to estimate $\mathbb {E}\left[ \, |Y(t, x) - Y(t, \widetilde{x})|^p \,\right]$ for $x, \widetilde{x} \in {{\mathbb {R}} ^N}$ and then to use Kolmogorov’s theorem to get the stated spatial regularity. To this end, we have that

3.16 $$\begin{aligned}&\mathbb {E}\left[ \, \left| I_1(t, x) - I_1(t, \widetilde{x}) \right| ^p \,\right] \nonumber \\&\quad \le \mathbb {E}\left[ \, \left( \int _0^t\int _{{\mathbb {R}} ^N} |w(x, y) - w(\widetilde{x}, y)||G(Y(s, y))|dyds \right) ^p \,\right] \nonumber \\&\quad \le C_G^pt^p \Vert w(x, \cdot ) - w(\widetilde{x}, \cdot )\Vert _{L^1({{\mathbb {R}} ^N})}^p\nonumber \\&\quad \le C_G^pt^pK^p_w |x-\widetilde{x}|^{p\alpha }, \end{aligned}$$

where we have used (**C3’**). Moreover, by Hölder’s and Burkhölder’s inequalities once again, we see that

$$\begin{aligned}&\mathbb {E}\left[ \, \left| I_2(t, x) - I_2(t, \widetilde{x}) \right| ^p \,\right] \nonumber \\&\quad \le 2^{p-1}\mathbb {E}\left[ \, \left| \int _0^t\int _{{{\mathbb {R}} ^N}}e^{-(t-s)}\left[ \sigma (Y(s, x))- \sigma (Y(s, \widetilde{x}))\right] \varphi (x - y)W(dyds)\right| ^p \,\right] \nonumber \\&\qquad + 2^{p-1}\mathbb {E}\left[ \, \left| \int _0^t\int _{{{\mathbb {R}} ^N}}e^{-(t-s)}\sigma (Y(s, \widetilde{x}))[\varphi (x- y) - \varphi (\widetilde{x}-y)]W(dyds)\right| ^p \,\right] \nonumber \\&\quad \le 2^{p-1}c_p\mathbb {E}\left[ \, \left( \int _0^t\int _{{{\mathbb {R}} ^N}}|\sigma (Y(s, x))- \sigma (Y(s, \widetilde{x}))|^2\left| \varphi (x - y)\right| ^2dyds\right) ^\frac{p}{2} \,\right] \nonumber \\&\qquad + 2^{p-1}c_p\mathbb {E}\left[ \, \left( \int _0^t\int _{{{\mathbb {R}} ^N}}\left| \sigma (Y(s, \widetilde{x}))\right| ^2|\varphi (x - y) - \varphi (\widetilde{x}-y)|^2dyds\right) ^\frac{p}{2} \,\right] \nonumber , \end{aligned}$$

for all $x, \widetilde{x} \in {{\mathbb {R}} ^N}$ and $p\ge 2$. Thus

3.17 $$\begin{aligned}&\mathbb {E}\left[ \, \left| I_2(t, x) - I_2(t, \widetilde{x}) \right| ^p \,\right] \nonumber \\&\quad \le 2^{p-1}c_pC_\sigma ^pt^{\frac{p}{2} -1}\Vert \varphi \Vert _{L^2({{\mathbb {R}} ^N})}^p\int _0^t\mathbb {E}\left[ \, |Y(s, x)- Y(s, \widetilde{x})|^p \,\right] ds \nonumber \\&\qquad + 2^{2(p-1)}c_pC_\sigma ^pt^{\frac{p}{2} }\Vert \varphi - \varvec{\tau }_{\widetilde{x}-x}(\varphi )\Vert _{L^2({{\mathbb {R}} ^N})}^p\left( 1 + \sup _{s\in [0, T], y\in {{\mathbb {R}} ^N}}\mathbb {E}\left[ \, \left| Y(s, y)\right| ^p \,\right] \right) , \end{aligned}$$

where we note that the right-hand side is finite thanks to Theorem 3.8. Returning to (3.15) and using estimates (3.16) and (3.17) we see that there exists a constant $C_T^{( p)}$ (depending on $T, p, C_G, K_w, C_\sigma , C_\varphi , \Vert \varphi \Vert _{L^2({{\mathbb {R}} ^N})}$ as well as $\sup _{s\in [0, T], y\in {{\mathbb {R}} ^N}}\mathbb {E}\left[ \, |Y(s, y)|^p \,\right]$), such that

$$\begin{aligned}&\mathbb {E}\left[ \, \left| Y(t, x) - Y(t, \widetilde{x}) \right| ^p \,\right] \nonumber \\&\quad \le C_T^{( p)}\left[ \mathbb {E}\left[ \, \left| Y_0(x) - Y_0(\widetilde{x}) \right| ^p \,\right] + |x - \widetilde{x}|^{p\alpha } + \int _0^t\mathbb {E}\left[ \, |Y(s, x)- Y(s, \widetilde{x})|^p \,\right] ds \right] \\&\quad \le C_T^{( p)}\left[ |x - \widetilde{x}|^{p\alpha } + \int _0^t\mathbb {E}\left[ \, |Y(s, x)- Y(s, \widetilde{x})|^p \,\right] ds \right] , \end{aligned}$$

where the last line follows from our assumptions on $Y_0$ and by adjusting the constant $C_T^{( p)}$. This bound holds for all $t\ge 0$, $x, \widetilde{x} \in {{\mathbb {R}} ^N}$ and $p\ge 2$. The proof is then completed using Gronwall’s inequality, and Kolmogorov’s continuity theorem once again.$\square$

## Comparison of the two approaches

The purpose of this section is to compare the two different approaches taken in Sects. 2 and 3 above to give sense to the stochastic neural field equation. Such a comparison of the two approaches in a general setting has existed for a long time in the probability literature [see for example Jetschke (1982, 1986), or more recently Dalang and Quer-Sardanyons (2011)], but we provide a proof of the main result (Theorem 4.1) in the Appendix for completeness.

Our starting point is the random field solution, given by Theorem 3.7. Suppose that the conditions of Theorem 3.7 are satisfied [i.e. $\varphi \in L^2({\mathbb {R}} ^N)$, $\sigma :{\mathbb {R}} \rightarrow {\mathbb {R}}$ Lipschitz, $G:{\mathbb {R}} \rightarrow {\mathbb {R}}$ Lipschitz and bounded, $w$ satisfies (**C2’**) and the given assumptions on the initial condition]. Then, by that result, there exists a unique random field $(Y(t, x))_{t\ge 0, x\in {\mathbb {R}} ^N}$ such that

4.1 $$\begin{aligned} Y(t, x)&= e^{-t}Y_0(x) + \int _0^te^{-(t-s)}\int _{{\mathbb {R}} ^N} w(x,y)G(Y(s,y))dyds \nonumber \\&\quad + \int _0^t\int _{{\mathbb {R}} ^N}e^{-(t-s)}\sigma (Y(s, x))\varphi (x-y)W(ds dy) \end{aligned}$$

where

4.2 $$\begin{aligned} \sup _{t\in [0, T], x\in {\mathbb {R}} ^N}{\mathbb {E}} \left[ |Y(t, x)|^2\right] < \infty , \end{aligned}$$

for all $T>0$, and we say that $(Y(t, x))_{t\ge 0, x\in {\mathbb {R}} ^N}$ is the random field solution to the stochastic neural field equation.

It turns out the that this random field solution is equivalent to the Hilbert space valued solution constructed in Sect. 2, in the following sense.

### **Theorem 4.1**

Suppose the conditions of Theorem 3.7 and Theorem 3.8 are satisfied. Moreover suppose that condition (**C1’**) is satisfied for some $\rho _w\in L^1({\mathbb {R}} ^N)$. Then the random field $(Y(t, x))_{t\ge 0}$ satisfying (4.1) and (4.2) is such that $(Y(t))_{t\ge 0} {:=} (Y(t, \cdot ))_{t\ge 0}$ is the unique $L^2({\mathbb {R}} ^N, \rho _w)$-valued solution to the stochastic evolution equation

4.3 $$\begin{aligned} dY(t) = [-Y(t) + \mathbf {F}(Y(s))]dt + \mathbf {B}(Y(t))dW(t),\quad t\in [0, T], \end{aligned}$$

constructed in Theorem 2.2, where $\mathbf {B}:H \rightarrow L_0(U, H)$ (with $U = L^2({\mathbb {R}} ^N)$ and $H= L^2({\mathbb {R}} ^N, \rho _w)$) is given by (2.14) i.e.

$$\begin{aligned} \mathbf {B}(h)(u)(x) {:=} \sigma (h(x))\int _{{\mathbb {R}} ^N}\varphi (x-y)u(y)dy, \quad h \in H,\ u\in U. \end{aligned}$$

### *Example 4.2*

We finish this section with an example illustrating the above result, and the applicability of the two approaches. Indeed, we make the same choices for the neural field kernel $w$ and noise term as in Bressloff and Webber (2012), by taking

$$\begin{aligned} w(x, y) = \frac{1}{2\beta }e^{-\frac{|x-y|}{\beta }}, \ x, y\in {\mathbb {R}} ^N, \quad \sigma (a) = \lambda a, \ a\in {\mathbb {R}} , \end{aligned}$$

where $\beta$ and $\lambda$ are constants. As noted in Sect. 2.6, $\beta$ determines the range of the local synaptic connections. Then, first of all, it is clear that condition (**C2’**) is satisfied (indeed $\Vert w(x-\cdot )\Vert _{L^1({\mathbb {R}} ^N)}$ is constant) and $\sigma$ is Lipschitz and of linear growth, so that (assuming the initial condition has finite moments), Theorems 3.7 and 3.8 can be applied to yield a unique random field solution $(Y(t, x))_{t\ge 0}$ to the stochastic neural field equation. Moreover, by Example 2 in Sect. 2.8, we also see that (**C1’**) is satisfied. Thus Theorem 2.2 can also be applied to construct a Hilbert space valued solution to the stochastic neural field equation (Eq. (4.3)). By Theorem 4.1, the solutions are equivalent.

## Conclusion

We have here explored two rigorous frameworks in which stochastic neural field equations can be studied in a mathematically precise fashion. Both these frameworks are useful in the mathematical neuroscience literature: the approach of using the theory of Hilbert space valued processes is adopted in Kuehn and Riedler (2014), while we the random field framework is more natural for Bressloff, Ermentrout and their associates in Bressloff and Webber (2012), Bressloff and Wilkerson (2012), Kilpatrick and Ermentrout (2013).

It turns out that the constructions are equivalent (see Sect. 4), when all the conditions are satisfied (which we emphasize is certainly the case for all usual modeling choices of the neural field kernel $w$ and noise terms made in the literature—see Sects. 2.6, 2.7 and Example 4.2). However, there are still some advantages and disadvantages for taking one approach over the other, depending on the purpose. For example, an advantage of the construction of a solution as a stochastic process taking values in a Hilbert space carried out in Sect. 2, is that it allows one to consider more general diffusion coefficients. Moreover, it easy to apply results from a large body of literature taking this approach (for example LDP results—see Remark 2.3). A disadvantage is that we have to be careful to impose conditions which control the behavior of the solution in space at infinity and guarantee the integrability of the solution. In particular we require that the connectivity function $w$ either satisfies the strong conditions (**C1**) and (**C2**), or the weaker but harder to check conditions (**C1’**) and (**C2’**).

On the other hand, the advantage of the random field approach developed in Sect. 3 is that one no longer needs to control what happens at infinity. We therefore require fewer conditions on the connectivity function $w$ to ensure the existence of a solution [(**C2’**) is sufficient—see Theorem 3.7]. Moreover, with this approach, it is easier to write down conditions that guarantee the existence of a solution that is continuous in both space and time (as opposed to the Hilbert space approach, where spatial regularity is somewhat hidden). However, in order to avoid non-physical distribution valued solutions, we had to impose a priori some extra spatial regularity on the noise (see Sect. 3.2).

## Appendix

### *Proof (of Theorem 4.1)*

The proof of the result involves some technical definition chasing, and in fact is contained in Dalang and Quer-Sardanyons (2011), though rather implicitly, of but see also Jetschke (1982, 1986). It is for this reason that we carry out the proof explicitly in our situation, by closely following Dalang and Quer sardanyons (2011 Proposition 4.10). The most important point is to relate the stochastic integrals that appear in the two different formulations of a solution. To this end, define

$$\begin{aligned} {\mathcal {I}} (t, x) {:= } \int _0^t\int _{{\mathbb {R}} ^N}e^{-(t-s)}\sigma (Y(s, x))\varphi (x-y)W(ds dy), \quad x\in {\mathbb {R}} ^N,\ t\ge 0, \end{aligned}$$

to be the Walsh integral that appears in the random field solution (4.1). Our aim is to show that

6.1 $$\begin{aligned} {\mathcal {I}} (t, \cdot ) = \int _0^te^{-(t-s)}\mathbf {B}(Y(s))dW(s), \end{aligned}$$

where the integral on the right-hand side is the $H$-valued stochastic integral which appears in the solution to (4.3).

Step 1: Adapting Proposition 2.6 of Dalang and Quer-Sardanyons (2011) very slightly, we have that the Walsh integral ${\mathcal {I}} (t, x)$ can be written as the integral with respect to the *cylindrical* Wiener process ${\mathcal {W}} = \{{\mathcal {W}} _t(u): t\ge 0, u\in U\}$ with covariance $\mathbf {Id}_U$.^8^ Precisely, we have

$$\begin{aligned} \mathcal {I}(t, x) = \int _0^t g^{t, x}_sd{\mathcal {W}} _s, \end{aligned}$$

for all $t\ge 0, x\in {\mathbb {R}} ^N$, where $g^{t,x}_s(y) {:=} e^{-(t-s)} \sigma (Y(s, x))\varphi (x-y)$, $y\in {\mathbb {R}} ^N$, which is in $L^2(\varOmega \times [0,T]; U)$ for any $T>0$ thanks to (4.2). By definition, the integral with respect to the cylindrical Wiener process ${\mathcal {W}}$ is given by

$$\begin{aligned} \int _0^t g^{t, x}_sd{\mathcal {W}} _s = \sum _{k=1}^\infty \int _0^t \langle g^{t, x}_s, e_k\rangle _Ud\beta _k(s), \end{aligned}$$

where $\{e_k\}_{k=1}^\infty$ is a complete orthonormal basis for $U$, and $(\beta _k(t))_{t\ge 0} {:=} ({\mathcal {W}} _t(e_k))_{t\ge 0}$ are independent real-valued Brownian motions. This series is convergent in $L^2(\varOmega )$.

Step 2: Fix arbitrary $T>0$. As in Section 3.5 of Dalang and Quer-Sardanyons (2011), we can consider the process $\{W(t), t\in [0, T]\}$ defined by

6.2 $$\begin{aligned} W(t) = \sum _{k=1}^\infty \beta _k(t) J(e_k) \end{aligned}$$

where $J:U\rightarrow U$ is a Hilbert-Schmidt operator.

$W(t)$ takes its values in $U$, where it is a $Q (= JJ^*)$-Wiener process with $\mathrm {Tr}(Q)<\infty$ [Proposition 3.6 of Dalang and Quer-Sardanyons (2011)]. We define $J(u) {:=} \sum _{k} \sqrt{\lambda _k}\langle u, e_k\rangle _Ue_k$ for a sequence of positive real numbers $(\lambda _k)_{k\ge 1}$ such that $\sum _k \lambda _k <\infty$.

Now define

$$\begin{aligned} \varPhi _s^{t,x}(u)&= \left\langle g^{t, x}_s, u \right\rangle _U, \end{aligned}$$

which takes values in ${\mathbb {R}}$. Proposition 3.10 of Dalang and Quer-Sardanyons (2011) tells us that the process $\{\varPhi _s^{t,x}, s\in [0, T]\}$ defines a predictable process with values in $L_2(U, {\mathbb {R}} )$ and

6.3 $$\begin{aligned} \int _0^t \varPhi _s^{t,x} dW(s) = \int _0^tg^{t, x}_sd{\mathcal {W}} _s, \end{aligned}$$

where the integral on the left is defined according to Sect. (2.2), with values in ${\mathbb {R}}$.

Step 3: We now note that the original Walsh integral ${\mathcal {I}} (\cdot , \cdot ) \in L^2(\varOmega \times [0, T]; H)$. Indeed, because of Burkhölder’s inequality with $p=2$,

$$\begin{aligned} \Vert {\mathcal {I}} \Vert _{ L^2(\varOmega \times [0, T]; H)}^2&= {\mathbb {E}} \left[ \int _0^T\Vert {\mathcal {I}} (t,\cdot )\Vert _H^2dt\right] = \int _0^T\int _{{\mathbb {R}} ^N}\mathbb {E}\left[ \, |{\mathcal {I}} (t,x)|^2 \,\right] \rho _w(x)dxdt\\&\le \Vert \varphi \Vert _{L^2({\mathbb {R}} ^N)}^2 \int _0^T\int _0^t\int _{{\mathbb {R}} ^N}e^{-2(t-s)}\mathbb {E}\left[ \, \sigma ^2(Y(s, x)) \,\right] ds \rho _w(x)dxdt, \end{aligned}$$

which is finite, again thanks to (4.2). Hence ${\mathcal {I}} (t, \cdot )$ takes values in $H$, and we can therefore write

$$\begin{aligned} {\mathcal {I}} (t, \cdot )&= \sum _{j=1}^\infty \langle {\mathcal {I}} (t, \cdot ), f_j \rangle _Hf_j= \sum _{j=1}^\infty \left\langle \int _0^t \varPhi _s^{t,\cdot } dW(s), f_j \right\rangle _Hf_j, \end{aligned}$$

by (6.3), where $\{f_j\}_{j=1}^\infty$ is a complete orthonormal basis in $H$. Moreover, by using (6.2)

6.4 $$\begin{aligned} {\mathcal {I}} (t, \cdot )&= \sum _{j=1}^\infty \left( \,\,\int _{{\mathbb {R}} ^N}\left( \,\,\int _0^t \varPhi _s^{t,x} dW(s)\right) f_j(x) \rho _w(x)dx\right) f_j\nonumber \\&= \sum _{j=1}^\infty \left( \,\,\int _{{\mathbb {R}} ^N}\left( \sum _{k=1}^\infty \int _0^t \varPhi _s^{t,x}(\sqrt{\lambda _k}e_k) d\beta _k(s)\right) f_j(x) \rho _w(x)dx\right) f_j. \end{aligned}$$

Finally, consider the $H$-valued stochastic integral

$$\begin{aligned} \int _0^te^{-(t-s)}\mathbf {B}(Y(s))dW(s), \end{aligned}$$

where $\mathbf {B}: H \rightarrow L_0(U, H)$ is given above. Then similarly

$$\begin{aligned}&\int _0^te^{-(t-s)}\mathbf {B}(Y(s))dW(s) = \sum _{j=1}^\infty \left\langle \int _0^te^{-(t-s)}\mathbf {B}(Y(s))dW(s), f_j\right\rangle _Hf_j\\&\qquad = \sum _{j=1}^\infty \left\langle \sum _{k=1}^\infty \int _0^te^{-(t-s)}\sqrt{\lambda _k}\mathbf {B}(Y(s))(e_k)d\beta _k(s), f_j\right\rangle _Hf_j\\&\qquad = \sum _{j=1}^\infty \left( \,\,\int _{{\mathbb {R}} ^N}\left( \sum _{k=1}^\infty \int _0^te^{-(t-s)}\sqrt{\lambda _k}\mathbf {B}(Y(s))(e_k)(x)d\beta _k(s)\right) f_j(x)\rho _w(x)dx\right) f_j. \end{aligned}$$

Here, by definition, for $x\in {\mathbb {R}} ^N$, $0\le s \le t$,

$$\begin{aligned} e^{-(t-s)}\sqrt{\lambda _k}\mathbf {B}(Y(s))(e_k)(x)&= \int _{{\mathbb {R}} ^N}e^{-(t-s)}\sigma (Y(s, x))\varphi (x-y)\sqrt{\lambda _k}e_k(y)dy \\&=e^{-(t-s)}\sigma (Y(s, x)) \langle \varphi (x\!-\!\cdot ),\sqrt{\lambda _k}e_k\rangle _U \!=\!\varPhi _s^{t,x}(\sqrt{\lambda _k}e_k), \end{aligned}$$

which proves (6.1) by comparison with (6.4).

Step 4: To conclude it suffices to note that the pathwise integrals in (4.1) and the $H$-valued solution to (4.3) coincide as elements of $H$. Indeed, it is clear that, by definition of $\mathbf {F}$,

$$\begin{aligned} \int _0^te^{-(t-s)}\int _{{\mathbb {R}} ^N} w(\cdot ,y)G(Y(s,y))dyds = \int _0^t e^{-(t-s)}\mathbf {F}(Y(s))ds, \end{aligned}$$

where the later in an element of $H$.$\square$

## Footnotes

1 The norm of $B \subset L_0(U,H)$ is classically defined as $\sup _{x \ne 0} \frac{\Vert Bx\Vert _H}{\Vert x\Vert _U}$.

2 The covariance operator $C:U\rightarrow U$ of $W$ is defined as ${\mathbb {E}} [\langle W(s),g\rangle _U \langle W(t),h \rangle _U]=s\wedge t\langle C g,h\rangle _U$ for all $g$, $h \in U$.

3 Technically this means that $\varPhi (s)$ is measurable with respect the $\sigma$-algebra generated by all left-continuous processes that are known at time $s$ when $(W(u))_{u\le s}$ is known (these process are said to be adapted to the filtration generated by $W$).

4 This would be for an infinite size cortex. The cortex is in effect of finite size but the spatial extents of $w_{loc}$ and $w_{lr}$ are very small with respect to this size and hence the model in which the cortex is ${\mathbb {R}} ^2$ is acceptable.

5 This can also be obtained by applying the operator $B$ to the representation (2.2) of $W$.

6 Recall that a collection of random variables $X = \{X(\theta )\}_{\theta \in \Theta }$ indexed by a set $\Theta$ is a Gaussian random field on $\Theta$ if $(X(\theta _1), \dots , X(\theta _k))$ is a $k$-dimensional Gaussian random vector for every $\theta _1, \dots , \theta _k\in \Theta$. It is characterized by its mean and covariance functions.

7 Precisely we consider functions $f$ such that $(t,x,\omega )\mapsto f(t,x,\omega )$ is measurable with respect to the $\sigma$-algebra generated by linear combinations of functions of the form $X(\omega )\mathbf{1 }_{(a, b]}(t)\mathbf{1 }_A(x)$, where $a, b \in {\mathbb {R}} ^+$, $A\in {\mathcal {B}} ({{\mathbb {R}} ^N})$, and $X:\varOmega \rightarrow {\mathbb {R}}$ is bounded and measurable with respect to the $\sigma$-algebra generated by $(W_s(A))_{s\le a, A\in {\mathcal {B}}({{\mathbb {R}} ^N})}$.
